# Riding the wave of innovation: immunoinformatics in fish disease control

**DOI:** 10.7717/peerj.16419

**Published:** 2023-12-08

**Authors:** Siti Aisyah Razali, Mohd Shahir Shamsir, Nur Farahin Ishak, Chen-Fei Low, Wan-Atirah Azemin

**Affiliations:** 1 Faculty of Science and Marine Environment, Universiti Malaysia Terengganu, Kuala Nerus, Terengganu, Malaysia; 2 Biological Security and Sustainability Research Interest Group (BIOSES), Universiti Malaysia Terengganu, Kuala Nerus, Terengganu, Malaysia; 3 Department of Biosciences, Faculty of Science, Universiti Teknologi Malaysia, Skudai, Johor, Malaysia; 4 Institute of Systems Biology (INBIOSIS), Universiti Kebangsaan Malaysia, Bangi, Selangor, Malaysia; 5 School of Biological Sciences, Universiti Sains Malaysia, Minden, Pulau Pinang, Malaysia

**Keywords:** Immunoinformatics, Computational biotechnology, Vaccines, Aquaculture, Fish diseases, Molecular dynamics simulation, In silico epitope-based vaccine design, Molecular docking, Adjuvant, Linker selection

## Abstract

The spread of infectious illnesses has been a significant factor restricting aquaculture production. To maximise aquatic animal health, vaccination tactics are very successful and cost-efficient for protecting fish and aquaculture animals against many disease pathogens. However, due to the increasing number of immunological cases and their complexity, it is impossible to manage, analyse, visualise, and interpret such data without the assistance of advanced computational techniques. Hence, the use of immunoinformatics tools is crucial, as they not only facilitate the management of massive amounts of data but also greatly contribute to the creation of fresh hypotheses regarding immune responses. In recent years, advances in biotechnology and immunoinformatics have opened up new research avenues for generating novel vaccines and enhancing existing vaccinations against outbreaks of infectious illnesses, thereby reducing aquaculture losses. This review focuses on understanding *in silico* epitope-based vaccine design, the creation of multi-epitope vaccines, the molecular interaction of immunogenic vaccines, and the application of immunoinformatics in fish disease based on the frequency of their application and reliable results. It is believed that it can bridge the gap between experimental and computational approaches and reduce the need for experimental research, so that only wet laboratory testing integrated with in silico techniques may yield highly promising results and be useful for the development of vaccines for fish.

## Introduction

Millions of farmers, food processors, traders, researchers, technical experts, and leaders all over the world are engaged in the daunting challenge of feeding a projected nine billion global population by 2050. Fish and other aquatic products from aquaculture play a significant role in meeting the dietary needs of all people, as well as the requirements of the poorest for food security (Mair et al., 2023). Aquaculture accounts for 49.2% of total aquaculture and fisheries production on a global scale, with proportions varying by region and production sector. Aquaculture is essential to meet the world’s need for fish for several reasons, including overfishing, habitat degradation, climate change, pollution, and unsustainable fishing practices (Divya & Devi, 2023). However, the sustainable development of the aquaculture sector is hindered by many factors, with the control of infectious diseases being one of the most significant challenges, as fish disease outbreaks have caused enormous economic losses in the aquaculture industry (Tavares-Dias & Martins, 2017; Peterman & Posadas, 2019; Fernández Sánchez et al., 2022; Abdelrahman et al., 2023). Losses in fish production, revenue, livelihoods, and international trade (citation) are major components of economic losses caused by fish disease outbreaks in aquaculture, emphasizing the need for effective fish disease management strategies.

Despite antibiotics or chemotherapeutics being used for fish disease treatment in aquaculture, drug resistance issues and safety concerns become obstacles to resolving fish disease outbreaks (Harikrishnan, Balasundaram & Heo, 2011; Sneeringer, Bowman & Clancy, 2019). Thus, fish vaccinations have been extensively employed in the aquaculture industry. Prior to deployment, fish vaccines, like those used in human and veterinary medicine, must pass stringent tests for safety and efficacy. In safety assessments, potential adverse effects on vaccinated fish, non-target species, and the environment are evaluated. These tests ensure that the vaccine does not cause excessive damage to the fish, has no negative effects on non-target organisms, and does not introduce harmful substances into the environment (Irshath et al., 2023). The effectiveness of fish vaccines is evaluated both in the laboratory and in the field. The immune response of the fish is monitored in the laboratory to corroborate that the vaccine induces an adequate immune response. Trials are conducted in the field to ensure that the vaccine provides protection against the targeted pathogen under real-world conditions.

Notably, although vaccines considerably reduce the likelihood of disease outbreaks, they do not guarantee complete immunity. Individual fish may respond differently to vaccination, similar to other animals, due to a variety of factors, including genetic variability, age, nutritional status, stress, and concurrent infections. This is why continuous monitoring of the efficacy and safety of vaccines is necessary (Zimmermann & Curtis, 2019). Vaccines are an essential component of the sustainable management of aquaculture, as they contribute to disease control and fish welfare while reducing the use of antibiotics. They provide a proactive and preventative approach to health management, which aligns with the overarching goal of assuring global food security. As is the case with all medications, the key to their successful application rests in their application in accordance with scientific research and established guidelines.

A fish vaccine typically contains a substance derived from pathogenic microorganisms in non-pathogenic forms that act as an antigen. By stimulating the fish’s immune system to combat a specific pathogen, the system is permitted to create a response, as well as a “memory” to cause the acceleration of the response, when the specific organism that causes the disease creates future infections (Yanong, 2017; Ma et al., 2019; Kayansamruaj, Areechon & Unajak, 2020). Traditional methods were used to develop a variety of vaccines, including killed whole-cell, live-attenuated, recombinant DNA, subunits, and toxoid vaccines (Gudding & Van Muiswinkel, 2013; Ma et al., 2019; Bouazzaoui et al., 2021). However, most authorized and commercial vaccines currently in use in the aquaculture industry are killed whole-cell vaccines while other vaccine groups are still being studied in live animals or are in the experimental phase (Adams, 2019; Mohd-Aris et al., 2019).

The killed whole-cell vaccine, also known as bacterin, is among the oldest vaccination technologies indigenously manufactured by many developing countries (Maeda et al., 2021). To make such a vaccine, it requires organisms that must be inactivated or that have died through physical or chemical procedures like inactivation with heat, irradiation with UV, or inactivation through formalin or chloroform (Lee et al., 2012; Tafalla, Bøgwald & Dalmo, 2013). When administered to the host, they induce strong protective humoral immune responses against those pathogens (Damodharan et al., 2021). Using killed whole-cell vaccines can prevent a number of viral disease outbreaks, which include infectious necrosis of the pancreas, spleen, and kidney; and pancreatic disease viruses; as well as bacterial diseases such as Vibriosis, enteric septicaemia of catfish, and Streptococcus infections (Assefa & Abunna, 2018; Ma et al., 2019).

Live-attenuated vaccines are types of vaccines that contain live microorganisms whose virulent properties were disabled under specific cultivation conditions to generate a broad immune response (Abdelhamed, Lawrence & Karsi, 2018; Heckman et al., 2022). Many scientific studies have focused on these vaccinations, which are being investigated for commercialization as fish vaccines due to their capacity to combat 209 infectious diseases caused by recognised and unknown pathogenic microorganisms still under investigation (Kayansamruaj, Areechon & Unajak, 2020). Unlike killed whole-cell vaccines, live-attenuated vaccines are able to induce both cell-mediated and humoral immune responses (Shoemaker et al., 2009; Côté-Gravel, Brouillette & Malouin, 2019). These vaccines with a minimum dosage are adequate to elicit long-lasting protective immune responses as they mimic the real infections caused by pathogens. This incident preferentially evokes T-cell proliferative responses relative to B-cell responses (Tajimi et al., 2019; Muñoz Atienza, Díaz-Rosales & Tafalla, 2021). Thus, they confer greater adaptive immune protection in fish compared with the induction of killed whole-cell vaccine or subunit vaccine (Sudheesh & Cain, 2017; Mohd-Aris et al., 2019). For instance, live-attenuated vaccinations have prevented herpesvirus disease (Dhar, Manna & Thomas Allnutt, 2014; Huang et al., 2021), columnaris disease (Shoemaker et al., 2011; Cai & Arias, 2021), and bacterial kidney disease (Evensen, 2016; Delghandi, El-Matbouli & Menanteau-Ledouble, 2020) caused by pathogens; KHV *Herpesvirus*, *Flavobacterium columnaris*, and *Renibacterium salmoninarum*, respectively.

The recombinant DNA vaccine is one of the experimental vaccines now in use and in research. Using the gene gun technique, the pathogen gene is cloned into the vector before being introduced into the host. Subsequently, the protein that functions as an antigen will be synthesised within the host and will elicit an immunological response (Lorenzen & LaPatra, 2005; Hølvold, Myhr & Dalmo, 2014; Collins, Lorenzen & Collet, 2019). Similar to live-attenuated vaccines, it induces both humoral and cellular immunity (Nascimento & Leite, 2012; Bedekar, Kole & Tripathi, 2020). For instance, Nile tilapia vaccinated with the recombinant DNA vaccine SL7207-pVAX1-sip had a higher survival rate following *Streptococcus agalactiae* infection (Zhu et al., 2017); while flounder fish were conferred with a protective immune response by administering a vaccine based on DNA that encoded the VAA gene of *Vibrio anguillarum* (Xing et al., 2019). This demonstrates that recombinant DNA vaccines are useful tools in investigating the key factor in the pathogenicity of the etiological agent to the fish and are economically viable in animals with extremely high value (Khan et al., 2016; Mzula et al., 2019).

When it is difficult to cultivate an organism, subunit vaccines are advantageous because they utilise the immunogenic component of the organism. Subunit vaccines may incorporate toxoids, subcellular fragments, and surface antigens. In comparison to inactivated, whole-organism vaccinations, these vaccines have limited immunogenicity. To enhance immunogenicity, adjuvants are necessary (Dadar et al., 2017). Many studies have reported that subunit vaccines such as recombinant subunit vaccines of grouper sleepy disease iridovirus (GSDIV) with montanide ISA could be utilised to decrease grouper mortality due to GSDIV infection (Mahardika et al., 2016). In addition, the efficacy of three different subunit vaccines against *Aeromonas salmonicida* infection in rainbow trout *Oncorhynchus mykiss* has been shown to significantly lower mortalities after 3 weeks (Marana et al., 2017). Although subunit vaccinations pose a relatively minimal risk of negative effects, retaining their antigen in their native form during the purification process may be difficult. Thus, organisms may be unable to detect antigens, resulting in these proteins failing to elicit an immune response in the host (Wang, Jiang & Wang, 2016; Abinaya & Viswanathan, 2021).

The composition of fish vaccines may differ from vaccines intended for human use with respect to the adjuvants and preservatives that are suitable for aquatic environments. Human vaccines are formulated with components safe for human use. Due to the differences in fish immune systems, comprehensive research and development are being conducted to formulate adjuvants to enhance subunit vaccines’ effectiveness in fish species. Meanwhile, human vaccines are subjected to extensive clinical trials prior to the approval, and the regulatory requirements for fish vaccines are specific to the aquaculture industry. Fish vaccines are calibrated to cater to the unique disease profiles and the needs of particular fish populations to ensure optimal health and protection against diseases.

Immunostimulants or adjuvants are routinely added to vaccinations containing inactivated pathogens or recombinant antigens to serve as vaccine carriers, thereby enhancing the vaccine’s efficacy and eliciting a powerful immune response (Tafalla, Bøgwald & Dalmo, 2013; Huang et al., 2014; Munangándu et al., 2020; Guo & Li, 2021). However, its effectiveness depends on the method of administration. There are three methods for administering vaccinations to fish: injection, immersion, and oral immunisation (Ringøet al., 2014; D’Amico et al., 2021). In general, injection is superior to oral delivery and immersion vaccination; however, this preference is dependent on the fish’s size (Embregts & Forlenza, 2016; Bøgwald & Dalmo, 2019). Notably, these approaches are only used on healthy fish because they are preventative and not curative (Wali & Balkhi, 2016; Miccoli et al., 2021). In general, the advantages and disadvantages of the common methods used to vaccinate fish could be summarized in Table 1. Overall, the selection of vaccination approach depends on various factors that include the fish species, types of vaccine, and the scale of the operation. A combination of different administration approaches or the use of different adjuvants and immunostimulants may be required to optimize the immune response and to ensure vaccine efficacy in disease prevention. Close monitoring is essential to evaluate the health status of the vaccinated fish population to identify potential adverse effects of the vaccines on fish health.

**Table 1:** Advantages and disadvantages of different vaccination methods that are common in fish farming.

Vaccine administration	Advantages	Disadvantages
Injection	• Controlled and precise dosage for optimal immune stimulation, reducing the risk of under or over-dosing. • Higher efficacy due to precise delivery of the vaccine directly into the fish, resulting in a potent immune response, thus, providing longer-lasting immune protection against the target pathogen.	• Induces stress due to the mechanical handling of fish during vaccination, which could potentially lead to negative physiological responses and reduced immunity. • Labor-intensive and time-consuming to vaccinate large numbers of fish. • Not suitable for small fish.
Immersion	• Non-invasive approach, thus, reducing stress and minimizing the risk of injury during vaccination. • Less labour-intensive, thus, cost-effective for large-scale aquaculture. • Time-efficient for mass vaccination of the cultured species.	Lower efficacy compared to the injection method due to the variations in vaccine uptake by different individuals, thus leading to inconsistent immune responses and protection levels in the vaccinated population of the cultured species.
Oral	• Non-invasive approach, and it is typically well-tolerated by fish, reducing stress and the potential risk of injury during vaccination. • Applicable to vaccinate small fish or fry. • Oral vaccines can be incorporated into fish feed, thus making it more practical for small- to large-scale fish farming.	• Variable uptake of the vaccines through fish digestive systems leads to inconsistent immune responses and protection levels in the vaccinated population of the cultured species. • Heat-sensitive vaccines could lose their efficacy during feed processing, storage, or digestion.

Nonetheless, commercial vaccine development is constrained by cost-effectiveness in the field. In comparison to terrestrial animals, fish require a higher antigen dose, therefore, developing cost-effective inactivated viral vaccines has proven problematic (Sommerset et al., 2005; Muktar & Tesfaye, 2016; Shefat, 2018). For example, live-attenuated vaccines require proper storage since they are live (Kumru et al., 2014; Prosser et al., 2021; Pambudi et al., 2022). Similarly, the killed whole-cell vaccine type involved a high manufacturing cost in cell culture tests, where a significant number of microorganisms are necessary to produce immunity, and the need for multiple injections may also exist depending on the distinguishing qualities of the vaccine (Vaughn, Whitehead & Durbin, 2009; Dias et al., 2013; Rodrigues & Plotkin, 2020). Moreover, when a recombinant DNA vaccine is used as an alternative, immunologic tolerance (hyporesponsive) may develop because the antigen is expressed in the host (Liu et al., 2011; Peignier & Parker, 2020) which renders the host incapable of mounting an immunological response following vaccination (Poolman & Borrow, 2011; Hobernik & Bros, 2018; Brisse et al., 2020). Furthermore, certain chemical treatments used in killed whole-cell vaccine development such as formaldehyde may alter antigenicity. This alteration necessitated the use of adjuvants in single or repeated doses to lessen the risk of antigenicity. Thus, this phenomenon not only raises production costs but also formulation and administration complexity (Furuya et al., 2010; Sanders, Koldijk & Schuitemaker, 2015; Martínez-Flores et al., 2021).

Certain fish species are too weak to withstand the stress induced by immunisation and may experience severe side effects after vaccination (Parra, Reyes-Lopez & Tort, 2015; Mugwanya et al., 2022). Moreover, it is difficult to analyse the relationship between pathogen and vaccine-induced immunity in fish species (Ángeles Esteban, 2012; Biller-Takahashi & Urbinati, 2014; Magadan, Sunyer & Boudinot, 2015; Smith, Rise & Christian, 2019a). Moreover, severe issues related to disease might develop at the stages of larvae or fry in other species, that is, prior to the organism growing sufficiently so that vaccination can occur or fully operational immune systems can form (Dhar, Manna & Thomas Allnutt, 2014; Muktar & Tesfaye, 2016; Hazreen-Nita et al., 2019). In this regard, the computational immunology technique, also known as the immunoinformatics approach, is one way that these limitations can be circumvented and overcome. Immunoinformatics bridges the gap between computer science and immunology by employing computational resources and methods to manage and comprehend immunology data. It contributes to the management of large datasets and aids in the creation of new hypotheses regarding immune responses (Tomar & De, 2014; Chatanaka et al., 2022; Wong et al., 2022).

While the practical implementation of immunoinformatics in aquaculture has been established, it is essential to recognise that there are significant differences between fish and human immune systems (Wang, Chen & Wang, 2019). Understanding these differences is crucial for the efficient application of immunoinformatics in the development of vaccines and treatments for fish. The innate immune system of fish, which is recognised as the first line of defence against a variety of pathogens, plays a more significant role than its homologue in mammals. Notably, primitive fish species with no jaws, such as lampreys and hagfish, have a profoundly different immune system than jawed vertebrates. This system lacks the typical B and T cells and Major Histocompatibility Complex (MHC) molecules observed in humans and advanced fish species. For adaptive immunity, these jawless species rely on a unique system of variable lymphocyte receptors (VLRs). Jawed fish, including cartilaginous (such as sharks) and bony (such as trout) species, have evolved B and T cells and MHC molecules, heralding the “modern” emergence of adaptive immunity (Buchmann, 2014; Mitchell & Criscitiello, 2020; Wang, Chen & Wang, 2019). Despite this, fish immune systems are less sophisticated than those of mammals, including humans. For instance, fish have fewer subsets of T cells, and their B cells are less diverse. The disparity also extends to MHC molecules. Fewer MHC class II molecules are present in fish than in humans, and their function is not as well understood. Some species, such as the Atlantic cod, lack MHC II molecules but possess a larger number of MHC I molecules (Boehm, Iwanami & Hess, 2012). Essentially, these differences highlight the need for predictive models and algorithms that are uniquely tailored to the immune response pathways of fish. Ideally, the data used to train these algorithms should be derived from the immune responses of fish. Taking into consideration these differences will permit the effective application of immunoinformatics to the development of more effective preventative and therapeutic measures for aquaculture health management.

Immunoinformatics has been applied in many research studies, particularly for disease prevention strategies, such as predicting immune cell populations, modelling immune responses, and studying autoimmune disorders and allergies. As our understanding of the immune system has increased in breadth and depth, this approach has naturally evolved to match this progression, giving rise to a term that encompasses this broader spectrum of activities. Immunoinformatics has emerged as a game-changing tool in the development of fish vaccines, addressing major obstacles and accelerating progress. Identification of immune epitopes within fish pathogens is one of the most important applications. Using immunoinformatics, researchers can effectively identify these epitopes: sections of the pathogen’s genetic sequences with the potential to elicit an immune response in fish (Forouharmehr et al., 2022a; Islam et al., 2022a; Islam et al., 2022b). This essential knowledge guides the development of vaccines based on epitopes, allowing for targeted immunisation that induces a specific protective immune response. In addition, immunoinformatics contributes substantially to the development of epitope-based vaccines by enabling scientists to select the most immunogenic and conserved epitopes (Forouharmehr et al., 2022b; Islam et al., 2022a). This strategic approach paves the way for the development of broad-spectrum vaccines, thereby protecting against multiple pathogen strains or variants.

The design of vaccines, the focus of this review, also plays a crucial role in zoonosis prevention. As the interface between humans and animals continues to evolve and become obscure, particularly in the context of aquaculture, the risk of zoonotic diseases those transmitted from animals to humans becomes more pressing. Immunoinformatics can be an indispensable tool for mitigating these hazards and safeguarding public health. The identification of pathogens at an early stage is a crucial application. This technique enables the detection of emergent viral, bacterial, or parasitic strains in fish populations prior to their posing a risk to humans through a combination of genomic sequencing and computational analysis. By identifying and characterising these potential zoonotic hazards in advance, measures can be implemented to prevent their spread and protect human populations. Immunoinformatics facilitates the development of vaccines that can act as barriers to disease transmission at its origin. These vaccines, designed for fish but effective against potential human pathogens, can control the disease in fish populations, thereby reducing the risk of human infection significantly. The same structural understanding and immune system interaction principles can be applied to the design of therapeutic drugs. These prospective treatments could combat pathogens that threaten both fish and human health, thereby serving dual purposes in zoonotic disease control.

This review aims to provide a comprehensive summary of immunoinformatics software that has been used in recent years which is essential for vaccine design, particularly in fish vaccine development. The vaccine design is explicated based on *in silico* epitopes, develop a multi-epitope vaccine, and investigate how immunogenic vaccines interact on a molecular level. Through this approach, the applied and valid results of the use of immunoinformatics to address diseases in fish are examined in relation to how frequently they occur. Additionally, immune mechanisms and immunoinformatics in fish disease are predicted using TLR signalling pathways and may draw the interest of pharmaceutical and synthetic immunologists in synthesizing and discovering the vaccine’s novel potential.

## Survey methodology

Using Web of Science, Scopus, PubMed, ScienceDirect, and Google Scholar, primary and secondary literature pertinent to this review’s topic was evaluated. These databases were used to search for the following terms: “fish diseases” and “aquaculture” in combination with, “immunoinformatics”, “computational biotechnology”, “bioinformatics”, “vaccines”, “Epitope prediction”, “T cell epitope”, “B cell epitopes”, “adjuvant”, “linker”, “Structural modelling”, “molecular docking”, “molecular dynamics simulation”, “immune mechanism”, “TLR signalling pathways”, “multi-epitope vaccine” along with using “+”, “AND”, and “OR” for a specific search result. The identified articles were initially examined for relevance to the topic and thoroughly read.

### *In silico* epitope-based vaccine design

#### Epitope prediction

**Figure 1:**
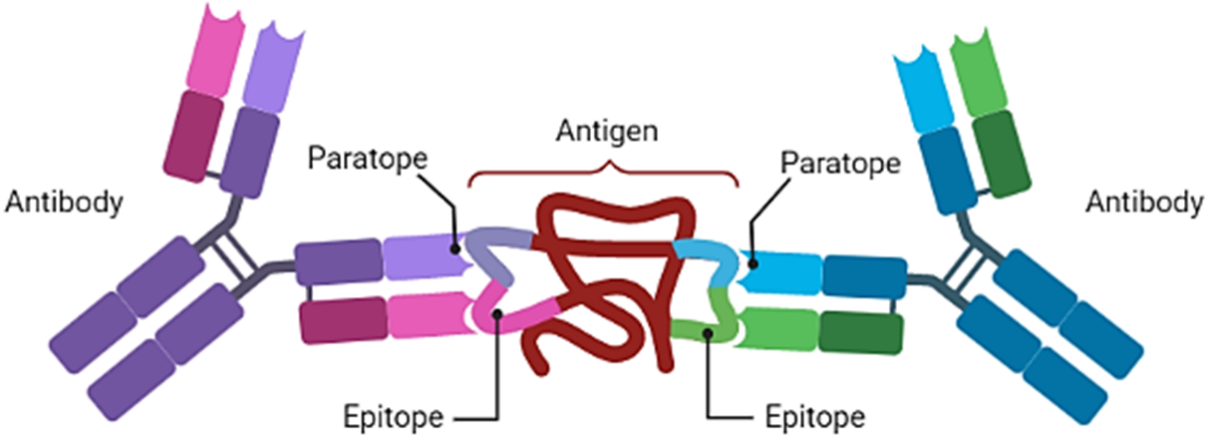
An antibody with two paratopes. These two paratopes are capable of binding to two pathogens. Non-covalent chemical interactions between epitopes and paratopes boost antigen–antibody binding. Created with BioRender.com.

By preventing and controlling viral illnesses in fish populations, the vaccination approach may help decrease the use of antibiotics in fish populations (Hoelzer et al., 2018; Ma et al., 2019). This is because the goal of vaccination is to stimulate the immune system so that it can form a long-lasting immunological memory and a stronger immune response when exposed to the pathogen during infections (Palgen et al., 2021). Therefore, the close relationship between immune system stimulation and the discovery of epitopes was demonstrated, which is a considerably interesting aspect when formulating vaccines to create efficacious epitope vaccines (Palatnik-de Sousa, Soares I da & Rosa, 2018). The design of vaccines based on epitopes required the antigenic peptides visible on the antigen-presenting cell (APC) and target cell surfaces to be identified (Dudek et al., 2010; Mugunthan & Harish, 2021). Antigens are any substances that induce immune systems to create antibodies to combat the issue and serve as sites of interactivity between the antibody, the B cells and T helper (T_H_) cells, as well as the molecules of the antigen. Such a site of interaction is referred to as an epitope (Marshall et al., 2018). Antibodies recognise antigens *via* interaction at the molecular level between paratopes (that is, the residues of the antibody implicated when binding occurs) and the interacting regions (epitopes) of the targeted molecules (antigens) (Jespersen et al., 2019), as illustrated in Fig. 1.

B-cell epitopes (BCEs) and T-cell epitopes (TCEs) are the two types of epitopes (TCEs). The B-cell epitope is a portion of an antigen that is connected to the immunoglobulin or antibody. B-cells recognise BCEs, which comprise a solvent area exposed to an antigen. Toxins and pathogens are neutralised by B-cell receptors (BCR), which are secreted or generated on their surface to target them with great specificity (antibodies) and thereby identify them for destruction (Sanchez-Trincado, Gomez-Perosanz & Reche, 2017; Bukhari et al., 2022). In the mapping of B-cell epitopes, predictors based on structures are becoming more popular because of the growing number of antibody-antigen complexes in the PDB and IMGT/3Dstructure-DB whose structures are three-dimensional (3D), in addition to the capacity for continuous and discontinuous epitopes (also called linear and conformational epitopes, respectively) to be anticipated (El-Manzalawy & Honavar, 2010; Soria-Guerra et al., 2015; Galanis et al., 2021). To predict B cell epitopes, the majority of the current approaches employ antigen amino acid sequences such as ABCpred (Malik et al., 2022), IEDB B-cell epitope tools (Vita et al., 2019), SVMTriP(Yao et al., 2012), BCPred (El-Manzalawy, Dobbs & Honavar, 2008), LBtope (Singh, Ansari & Raghava, 2013), and BepiPred 2.0 (Jespersen et al., 2017). Meanwhile, the prediction of conformational B cell epitopes has involved various approaches, such as DiscoTope−2.0, BEpro (formerly known as PEPITO) (Sweredoski & Baldi, 2008), ElliPro (Ponomarenko et al., 2008), EPCES (Liang et al., 2009), EPSVR (Liang et al., 2010), EPMeta (Liang et al., 2010), Epitopia (Rubinstein et al., 2009) and SEPPA (Sun et al., 2009).

**Figure 2:**
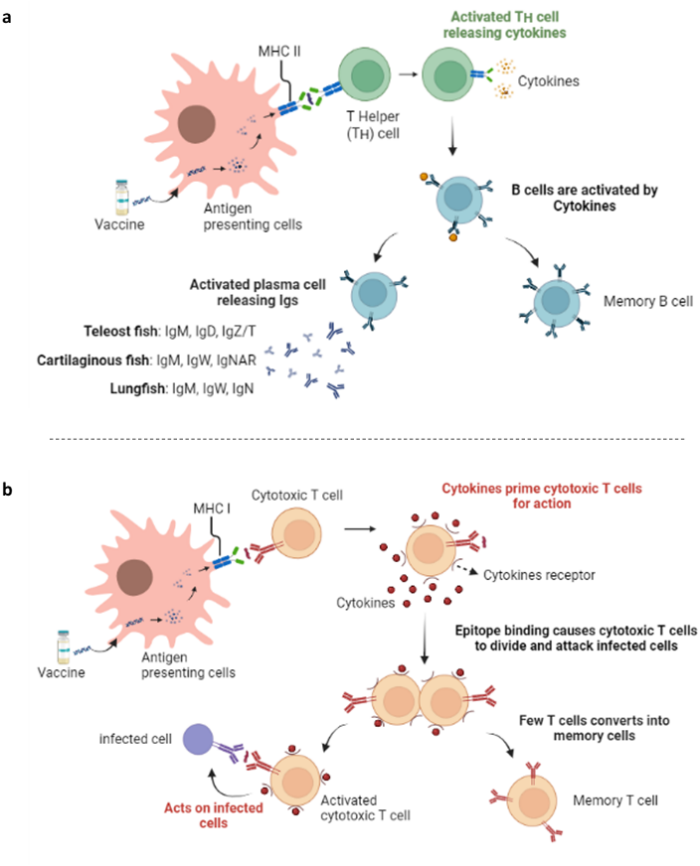
Immunological basis of the fish vaccine. (A) Humoral immune response. (B) Cell-mediated immune response. Created with BioRender.com.

In contrast, a T-cell epitope is a peptide obtained *via* an antigen. They can be recognised by particular receptors named T-cell receptors (TCR) when they bind to key histocompatibility complex (MHC) molecules that appear on the surfaces of APC cells (Sharma & Holt, 2014; Bukhari et al., 2022). TCEs in complex with MHC proteins are recognised by two group subsets of T cells *i.e.,* T helper (T_H_) or CD4^+^ T cells and cytotoxic T lymphocytes (CTL) or CD8^+^ T cells with different functionality (Wieczorek et al., 2017; Marshall et al., 2018). A T_H_ cell response is produced when TCRs on CD4^+^ T cells become bound to MHC class II–peptide complexes, which are frequently created in a professionally made APC. In contrast, CTL responses are elicited when TCRs on CD8^+^ T cells can fix to MHC class I–peptide complexes that nucleated cells present (Lundegaard, Lund & Nielsen, 2012; Sanchez-Trincado, Gomez-Perosanz & Reche, 2017). Cytotoxic T lymphocytes (CTL) that have been activated produce cytokines, which cause them to divide and destroy the infected cells. Similarly, several of them transform into memory T cells (Kar et al., 2020) (Fig. 2B). Similarly, active cytokines cause B-cells to develop into plasma cells and memory B cells. Consequently, the activated plasma cell or B-cell releases antibodies or immunoglobulins (Igs) that are responsible for clearing the infection. Type of Igs also differ in the bony fish group, such as teleost fish (IgM, IgD, and IgZ/T), cartilaginous fish (IgM, IgW, IgNAR), and lungfish (IgM, IgW, IgN) (Smith, Rise & Christian, 2019) (Fig. 2A). This fact indicates that the strength of the MHC molecule’s epitope binding is a key factor in determining T-cell epitope immunogenicity (Mahendran et al., 2016a; Ogishi & Yotsuyanagi, 2019).

The three crucial stages of immunogenicity in T-cell epitopes are as follows: antigens are processed, peptides attach to an MHC molecule, and cognate TCRs recognise this. When determining a TCE, MHC-peptide binding utilises the greatest selectivity of the three steps. As a result, the primary basis for anticipating TCEs is peptide-MHC binding prediction (Sanchez-Trincado, Gomez-Perosanz & Reche, 2017; Antunes et al., 2018; Feng, Zeng & Ma, 2021). Numerous computational methods have been investigated and reviewed to predict TCEs and MHC-binding peptides, wherein both are computed based on binding matrices, binding motifs, decision trees, artificial neural networks (ANN), hidden Markov models (HMM), support vector machines (SVM), homology modelling and protein docking techniques as well as quantitative structure–activity relationship (QSAR) analysis (Tong & Ren, 2009; Fleri et al., 2017; Kar et al., 2018). HTL and CTL epitopes can be predicted using a variety of current bioinformatics tools, such as the IEDB database, RANKPEP server (Reche et al., 2004), ProPred (Singh & Raghava, 2001; Reynisson et al., 2020), NetMHCIIpan 3.2 (Jensen et al., 2018), NetCTL−1.2 (Larsen et al., 2007), ProPred1 (Singh & Raghava, 2003), NetMHCpan−4.1 (Reynisson et al., 2020), MHCpred 2.0 (Guan et al., 2006), EpiJen (Doytchinova, Guan & Flower, 2006), CTLPred and Expitope (Haase et al., 2015).

Although the majority of the B- and T-cell prediction tools have been developed and trained using data derived from human and mammalian major histocompatibility complex (MHC) or human leukocyte antigen (HLA) alleles, these tools are still relevant and applicable in vaccine design for aquaculture species. Despite the differences in MHC and HLA alleles between human and fish species, it is noteworthy that the similarities in the immune mechanisms involving B- and T-cell responses are crucial in the selection of antigens for vaccine design. The specific MHC for antigen presentation differ between human and fish species, but the conserved regions in antigens contribute to stimulating cross-reactive immune responses across species. The prediction tools are able to identify epitopes within these conserved regions, which could be recognized by fish immune cells and eventually lead to a specific immune response against the target pathogen. In addition, the functional similarities of immune cell receptors (B- and T-cell receptors) between fish and human enables the application of these tools to predict potential epitopes that are immunogenic in fish, based on the knowledge of B- and T-cell receptors in human. These tools have been utilized and demonstrated success in predicting epitopes for vaccine design against tilapia lake virus (Islam et al., 2022a) and *Edwardsiella ictaluri* in Nile tilapia (Machimbirike et al., 2022a), *Streptococcus iniae* (Forouharmehr et al., 2022a), *Flavobacterium columnare* (Mahendran et al., 2016b), and against *Ichthyophthirius multifiliis* (Ghosh et al., 2023). Nonetheless, experimental validation studies are indispensable. Candidate epitopes predicted by the immunoinformatics tools can be chemically synthesized and tested *in vitro* or *in vivo* to evaluate the immunogenicity and efficacy of immune activation in fish.

### Construction of multi-epitope vaccine

#### Adjuvant selection

Peptide-based vaccinations, also known as epitope vaccines, are potential immunotherapeutic options and have been shown to have considerable advantages over conventional vaccines in multiple studies. However, when utilised alone in vaccine design, epitope vaccines are linked with low protection, which can be overcome by conjugating antigenic epitopes with adjuvants and helper peptides. Several roles for vaccine adjuvants have been proposed; (i) dose-sparing strategy; (ii) accelerating seroconversion rates by enhancing antibody and cell-mediated immune responses; (iii) diversifying the adaptive immune profile and (iv) enhancing vaccine production by using smaller amounts of antigen (Honda-Okubo, Baldwin & Petrovsky, 2021; Lemoine et al., 2021). Toll-like receptor (TLR) agonists, antimicrobial peptides and helper peptides are the most common adjuvants used in peptide-based vaccine construction (Shanmugam et al., 2012; Gupta et al., 2020). TLRs also known as pattern recognition receptors, recognise common surface antigens found on microbes and act as a bridge between innate and adaptive immunity. In addition to TLR agonists, helper peptides and adjuvants derived from bacteria are used to boost the immune effects of peptide-based vaccines, including PADRE, Hsp70, *β*-defensin, bacterial toxins, cell wall components, flagellin, lipopolysaccharides (LPS), nucleic acids, and CpG oligodeoxynucleotides (ODN) (Gries et al., 2019; Wang, Chen & Wang, 2019; Wangkahart, Secombes & Wang, 2019; Liang et al., 2020).

Using bioinformatics in vaccine research and development has allowed for improved vaccination formulations and adjuvant selection. The development of adjuvants can be guided by databases including information on PRRs and their ligands. Numerous databases can be employed to select adjuvants, including:

1. Vaxjo (https://violinet.org/vaxjo/) is a web-based vaccine adjuvant database that includes approximately 400 vaccines that use an adjuvant against over 80 pathogens, cancers, or allergies (Sayers et al., 2012).
2. VaccineDA (Vaccine DNA adjuvants). This web-based resource (https://webs.iiitd.edu.in/raghava/vaccineda/) was developed to design immunomodulatory oligodeoxynucleotides (IMODN) -based vaccine adjuvants (Nagpal et al., 2015).
3. imRNA (https://webs.iiitd.edu.in/raghava/imrna/) is used to predict and design potential immunomodulatory RNA-based vaccine adjuvants (Chaudhary et al., 2016).
4. VaxinPAD (https://webs.iiitd.edu.in/raghava/vaxinpad/) employs SVM-based models to design peptide-based vaccines and allows users to perform virtual screenings that incorporate data from experimentally validated immunomodulatory peptides (Nagpal et al., 2018).

#### Linker selection

Linkers, also known as ‘spacers’, are essential components in the design of multi-epitope vaccines (MEV) or peptide-based vaccines. They are critical for interdomain interactions, structural stability and functionality of vaccines. Fusion of epitopes without suitable linkers can result in negative outcomes such as 3D structural misfolding, low yield in vaccine production and bioactivity impairment. Despite their importance in recombinant MEV technology, the selection and rational design of linkers have not yet been thoroughly investigated. Flexible, rigid, and cleavable linkers are the three groups into which structural linkers can be categorised. To ensure flexibility of movement and interactivity between associated protein domains, those in the first linker group contain high levels of small and hydrophilic amino acids like glycine and serine. In contrast to flexible linkers, rigid linkers may be utilised more effectively to maintain the desired stability or bioactivity between fusion protein domains. Multiple alanine and proline residues in these linkers exhibit a stiff structure that reduces interactions and separates the functional domains of the designed antigens (Gräwe et al., 2020). Cleavage linkers, on the other hand, are utilised to divide domains or peptides by proteolytic cleavage in order to decrease steric hindrance and achieve the independent biological function of a single domain when the linker is cleaved (Leung et al., 2020; Poreba, 2020)

Linker selection is influenced by the amino acid arrangement, length, hydrophobicity, secondary structure, and potential interaction with other immunogenic construct components. Linker DB which was created by Integrative Bioinformatics VU (IBIVU) at the Vrije Universiteit of Amsterdam, is the most recent database of linker peptides that enables the selection of prospective linkers for novel fusion proteins. This system provided a list of possible linkers based on the user-searched linker length, solvent accessibility, sequence motif, and protein source. Choosing which criteria to apply enables the user to opt for the linkers they require, depending on the conformation, adaptability, and stability needed to ensure the proteins function biologically in their natural environments.

#### Prediction of vaccine antigenicity, allergenicity, toxicity and physicochemical properties

When designing and developing efficacious and safe candidates to use as vaccines, the vaccine constructs need to contain robust antigenicity but retain low toxic and allergenic levels. With advances in peptide synthesis, it is now feasible to fine-tune the physicochemical characteristics of peptides by including significant biochemical changes, maximising peptide functionality to reduce toxicity and allergenicity without limiting therapeutic effectiveness. A constructed vaccine sequence’s physicochemical properties are often determined using the PortParam server (https://web.expasy.org/protparam/), which can compute the composition of amino acids, molecular weights (Mw), isoelectric points (pI), instability indices, predicted half-lives, and grand averages of hydropathicity (GRAVY). The isoelectric focusing approach can be employed to induce the isoelectric point (pI) computations to create buffer systems to purify proteins. Stability is predicted to apply to proteins when their instability index is below 40. A protein’s aliphatic index (AI) refers to the relative volume taken up by its aliphatic side chain, which features, for instance, the amino acids alanine, valine, isoleucine, and leucine. Higher AI values will increase the thermal stability of globular proteins across a wider temperature range. A lower GRAVY value indicates a hydrophilicity pattern that is better suited for interaction with water.

The most significant requirement for efficient protein design is the antigenicity of the vaccine candidate, a high antigenicity score is expected to result in a greater immune response. A protein’s antigenicity could be predicted by a range of servers, including Vaxijen (http://www.ddg-pharmfac.net/vaxijen/VaxiJen/VaxiJen.html) and ANTIGENpro (http://scratch.proteomics.ics.uci.edu/). The former method of predicting antigens is free from alignment and utilises the transformation of protein sequences through auto cross-covariance (ACC) to form uniformly developed vectors of the key attributes of amino acids, thus circumventing the limitations of methods that depend on alignment (Flower et al., 2017). It was designed to allow antigen categorization exclusively based on protein physicochemical characteristics, without relying on sequence alignment. ANTIGENpro employs a pathogen-independent and sequence-based approach for predicting protein antigenicity (Magnan et al., 2010). This server predicts the entire protein antigenicity and the algorithm is trained to utilise reactivity data collected from protein microarray analysis for five pathogens including fungi, parasites, viruses, bacteria and tumours.

Toxicity is the ability of a material to cause damage in a live organism by destabilising and interfering with normal cellular activity. The ToxinPred tool (https://crdd.osdd.net/raghava/toxinpred/) can predict the toxicity of the computationally produced vaccine. ToxinPred is a support vector machine-based (SVM) approach to predicting peptide toxicity from sequence information using a position-specific scoring algorithm. ToxinPred was trained on a collection of known toxic and non-toxic peptides from the Universal Protein Resource (Gupta et al., 2013). The Toxins and Toxins Target Database (T3DB) which combines over 42,000 toxin data points with extensive toxin target information, is another resource that can be used to predict the toxicity of a vaccine candidate. It predicts if the constructed peptide vaccine may induce hypersensitivity responses. To determine the allergenicity of the potent vaccine, several services, such as AllerCatPro (Maurer-Stroh et al., 2019), AlgPred (Sharma et al., 2021), and AllerTop (Dimitrov et al., 2014) can be used to identify the allergenicity of the potent vaccine. In the former method, a protein’s allergenic potential is predicted through its three-dimensional structure and the similarity of its sequence of amino acids to the data in a library of identified protein allergens. AlgPred allows the prediction of allergen using multiple allergenicity prediction approaches based on IgE epitope mapping, MAST motif alignment, allergen-representative peptides (ARPs) BLAST, support vector machines, and hybrid approaches. Finally, AllerTOP−2.0 employs a technique that is based on a protein’s physicochemical similarity to known allergens.

#### Structural modelling, assessment, and validation

A strategy based on structures does not rely exclusively on data from binding and information about sequences; instead, it leverages structural data and computation-based approaches created in structural biology so that binders of potential suitability can be identified. Vaccine sequences are received by a website that predicts protein structures so that three-dimensional structure models can be created once the incorporation of BCEs and TCEs has occurred by utilising suitable linkers and intramolecular adjuvants. The molecular structure of MHC molecules and their interactions with peptides can be utilised to create complex 3D models with other peptides, aiding in the explanation of atomistic aspects of molecular structures connected to biological system operation.

Emerging developments in machine learning and deep learning offer unprecedented opportunities for aquaculture, particularly in the development of effective fish vaccines. These innovations could be used to devise better vaccines for fish. AlphaFold uses deep learning models to predict the 3D structures of proteins based on their amino acid sequences. It has tremendous ramifications in fish health management and aquaculture that cannot be understated as we move from theoretical to practical applications. This instrument enables researchers to navigate the complexity of protein structures, which serve as the blueprint for comprehending the antigen-antibody interaction, the linchpin of the immune response. Alphafold can predict the structure of several outer membrane proteins (OMPs), such as monomeric outer membrane protein A (OmpA), outer membrane protein 34 (Omp34), and a nucleoside-specific outer membrane transporter protein Tsx (OmpTsx) from rainbow trout (*Oncorhynchus mykiss*) afflicted with *Acinetobacter johnsonii* (Bi et al., 2023). This demonstrates the importance of AlphaFold in the design of subunit vaccines, which could exploit structural similarities in proteins across pathogens to enhance pathogen resistance in aquaculture environments. AlphaFold emerges as a potent instrument that revolutionises the landscape of vaccine development in aquaculture. With accurate protein structure prediction, we can accelerate the identification of potential vaccine targets and the development of effective treatments for diseases that threaten aquaculture production. With a reliable structural model, researchers can design vaccines that expose these epitopes to the fish immune system in a highly specific manner, thereby increasing the probability of a robust immune response.

Similarly, RosettaFold uses machine learning techniques to predict protein structures and could play a comparable role in the development of aquaculture vaccines. The precise prediction of the structures of proteins associated with fish diseases could assist in the development of multi-epitope vaccines. Such vaccines would target multiple proteins or multiple parts of a pathogen’s protein, evoking a more robust immune response. This is especially important when considering the enormous variety of pathogens that can affect fish and the inherent difficulty of designing vaccines that provide protection for a variety of fish species. Utilising these sophisticated predictive tools could usher in a new era of fish vaccine development, resulting in not only more effective but also more cost-effective vaccines. This would considerably improve disease resistance and sustainability within the aquaculture industry, which is essential given the increasing reliance on aquaculture for food production worldwide. However, despite the promising potential of these tools, it is essential to note that their outputs are computational predictions. Consequently, any vaccines developed based on these predictions must undergo rigorous laboratory testing and clinical trials to ensure their safety and effectiveness. In addition, ongoing research and refinement of these computational tools will be necessary to improve their predictive accuracy, thereby maximising their utility in furthering aquaculture vaccine design.

**Figure 3:**
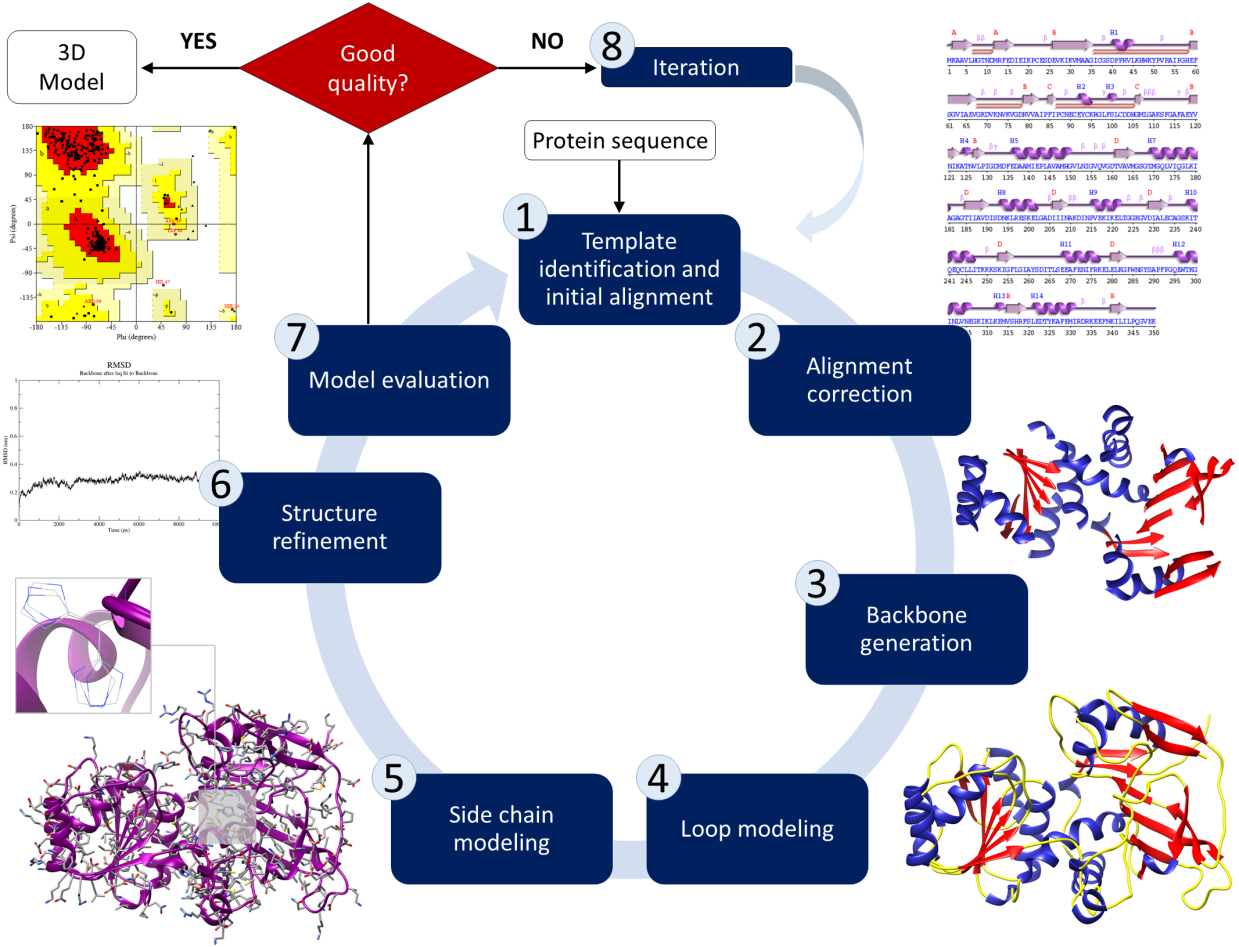
Schematic illustration of the basic process of comparative modelling for protein structure prediction. This method consists of several steps including template identification, initial alignment, alignment correction, backbone generation, loop and side-chain modelling, structure refinement and model evaluation.

Predicting protein tertiary structure can be accomplished using one of three methods: (1) Homology modelling, (2) threading, and (3) *ab initio* prediction. This method involves searching the Protein Data Bank (PDB) for structure-based similarities in the sequencing of the anticipated domains, from which a set of matches is generated according to E-values, alignment lengths, identities, and total scores. The basis for comparative modelling, which can be referred to as homology modelling, is the connection between target sequences and no fewer than one recognised three-dimensional structure belonging to the same family. Proteins that are aligned and have a higher percentage of identical residues imply evolutionary relationships. This method consists of several steps: (1) template identification and initial alignment, (2) alignment correction (3) backbone generation (4) loop and side-chain modelling, and (5) structure refinement and model evaluation (Fig. 3). Homology modelling is considered the most reliable method for predicting a protein’s structure. Nevertheless, it can be complicated to identify suitable template structures that have high coverage of sequences and sequence identities. In general, template structures with a coverage of less than 35% are considered unreliable templates. EasyModeller 4.0 (Kuntal, Aparoy & Reddanna, 2010), SWISS-MODEL (Waterhouse et al., 2018), Rosetta (Leman et al., 2020), and Phyre2 (Kelley et al., 2015) are the most often used internet servers for homology modelling.

Threading, also known as fold recognition, is an alternative method if homology modelling cannot be applied. By comparing a template sequence to a collection of structural folds, this approach returns a list of scores. A known peptide–MHC complex structure is utilised to predict the binding structures of other peptides to the same MHC molecule by maximising the alignment of the amino acid sequence and their 3D structural patterns. I-TASSER (MacCarthy et al., 2022), Phyre2 (Kelley et al., 2015), and RaptorX (Wang et al., 2016) servers are used for threading modelling. *Ab initio* protein modelling predicts 3D structures based on novel folds and can be utilised if the structure of interest is unavailable or if the sequence identity between the template and the protein of interest is less than 30%. Based on physical principles, this method involves computing all energy parameters of protein folding and determining the state with the lowest free energy. ROBETTA (Park et al., 2018), TrRosetta (Du et al., 2021) and I-TASSER (MacCarthy et al., 2022) are servers that can be used to predict the *ab initio* protein structure.

**Figure 4:**
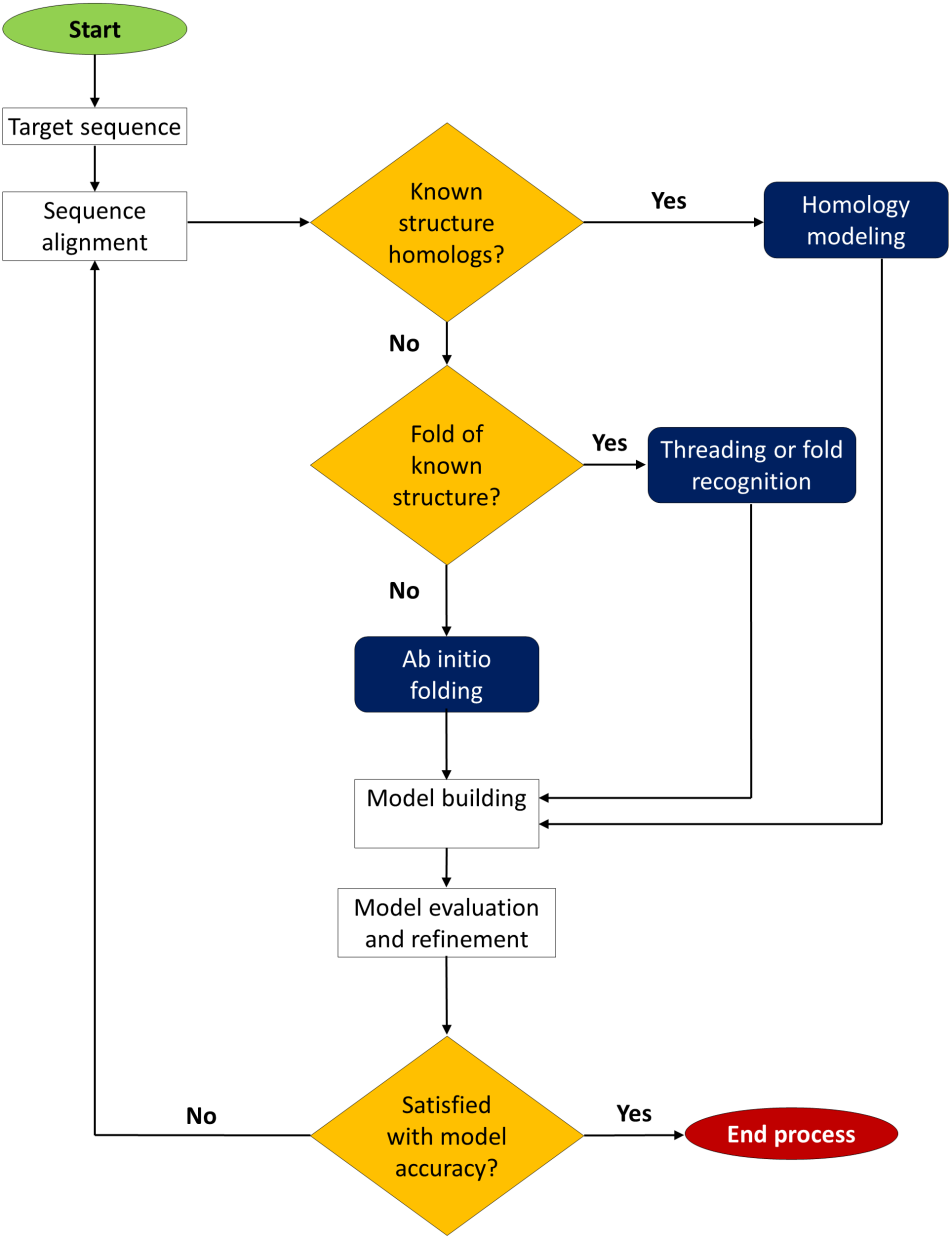
Decision-making chart for protein structure prediction method. The prediction of the 3D structure of a protein can be carried out with one of these three approaches: homology modelling, threading, or *ab initio* prediction.

Structural validation is an important step in protein modelling to assure the quality of the models. Energy minimisation and structural refinement are applied to each model to enhance the three-dimensional structures’ quality so that structural errors and steric conflicts in the structure of each protein can be eliminated. Several structure validation tools (Razali et al., 2016; Razali & Shamsir, 2020) such as PROCHECK (http://www.ebi.ac.uk/thornton-srv/software/PROCHECK/), Verify3D and ERRAT (https://saves.mbi.ucla.edu/) can be used to assess model quality before and after refining. PROCHECK provides a Ramachandran plot that calculates phi–psi torsion angles for each residue and analyses the overall stereochemical quality of 3D structures of protein models. Verify3D evaluate the compatibility of an atomic model (3D) with its amino acid sequence (1D) while the ERRAT server assesses the overall quality factor for nonbonded atomic interactions. The model quality assessment is critical for determining the overall correctness of the structure as well as the local accuracy of each protein fragment. Selecting the best protein modelling approach is dependent on the availability of known homologues, the folds of known structures, and the quality of 3D structures. Figure 4 depicts a schematic illustration of protein structure decision-making prediction based on several modelling methodologies.

In addition to the advancements made in protein structure prediction, the development of self-assembling immunogens is also making headway in the vaccine research landscape.

Self-assembling immunogens, such as virus-like particles (VLPs) and self-assembling protein nanoparticles (SAPNs), are used in the development of fish vaccines to enhance their immunogenicity and stability (Ma et al., 2019; Abudula et al., 2020; Nakahira et al., 2021). These immunogens are formed from the self-assembly of viral or bacterial proteins, which mimic the shape and size of native virions. Self-assembling peptides can also act as adjuvants themselves by forming an antigen depot, directing vaccines to antigen-presenting cells (APCs), and enhancing immune-cell priming (Abudula et al., 2020). These self-assembling structures, often designed as nanoparticles, mimic the native size and shape of viruses or bacteria and present multiple copies of an antigen or epitope on their surface. This form of antigen presentation stimulates a robust immune response by imitating the repetitive antigenic patterns of many pathogens. The use of self-assembling immunogens in fish vaccines offers several advantages, including precise antigen display, enhanced immunogenicity, and stability (Rudra et al., 2010).

Within the field of aquaculture, the potential benefits of self-assembling immunogens are multifaceted. Primarily, these structures could significantly enhance the immune response in fish. Due to the repetitive, ordered array of antigens that these nanoparticle vaccines present, they can stimulate the immune system more effectively, leading to a more potent and enduring response than traditional vaccines. Moreover, the versatility of self-assembling immunogens opens doors to designing broad-spectrum or multivalent vaccines. Such vaccines could potentially combat multiple strains or species of pathogens, addressing the substantial challenge of pathogenic diversity in aquaculture. On a practical note, self-assembling immunogens might present advantages in terms of stability and scalability. Assuming the initial design and production processes are fine-tuned, these vaccines could potentially be produced on a large scale and might exhibit enhanced stability under a variety of environmental conditions, an essential consideration for aquaculture operations worldwide. However, the development of self-assembling immunogens for fish vaccines is not without challenges. The design and production processes for these vaccines can be complex, necessitating further research to streamline them. Furthermore, experimental validation of the safety and efficacy of these vaccines in target fish species remains paramount. In this context, computational methods could play a significant role.

Self-assembling immunogens can be developed using computational methods such as AlphaFold and RosettaFold (Castro et al., 2022; Morales-Hernández, Ugidos-Damboriena & López-Sagaseta, 2022; Olshefsky et al., 2022). These computational methods enable the design of self-assembling immunogens by predicting the structure of proteins and their interactions with other proteins. AlphaFold uses deep neural networks to predict protein structures with high accuracy, while RosettaFold uses a combination of computational methods to predict protein structures. These computational methods can be used to design self-assembling immunogens with specific epitopes or antigens, which can enhance their immunogenicity and specificity.

The use of computational methods in the development of self-assembling immunogens offers several advantages, including the ability to design immunogens with specific properties and the ability to optimise immunogenicity and stability (Castro et al., 2022; Morales-Hernández, Ugidos-Damboriena & López-Sagaseta, 2022; Olshefsky et al., 2022). These tools could help design self-assembling immunogens by predicting the structures of antigens and then guiding the design of self-assembling proteins or peptides that best present these antigens. Overall, self-assembling immunogens provide a promising avenue for fish vaccine development. Their potential, coupled with the burgeoning capabilities of machine learning and deep learning tools, underscores an exciting frontier in aquaculture vaccine design. Nevertheless, it is crucial to maintain a balance between this optimism and a realistic understanding of the extensive validation and optimisation these novel approaches require.

### Molecular interaction of immunogenic vaccine

#### Molecular docking and molecular dynamics simulation

Protein-peptide docking is another important tool for predicting the efficacy of a vaccine. Unlike the costly and lengthy approaches involved in crystallising and structurally resolving a TCR-MHC complex, the computational tool of molecular docking can efficiently and cost-effectively enable intermolecular-level interactions within ligand–receptor complexes to be studied. This prediction is made using a software that includes (1) regeneration of all possible ligand structure formations, (2) placement of all ligand formations in a cavity of the active target protein position, and (3) scoring function based on free energy or binding energy. For molecular docking analysis, numerous software and tools have been created, including standalone applications such as Autodock Vina (Eberhardt et al., 2021), Autodock 4 (Santos-Martins et al., 2021), ZDOCK (Vreven et al., 2020), Glide (Alogheli et al., 2017) and GOLD (Martin et al., 2020). Several online servers, such as RosettaDock (Marze et al., 2018), ClusPro (Alekseenko et al., 2020), and HADDOCK (Roel-Touris et al., 2019) are also available to study protein-protein docking interactions. However, these web servers are not suitable for large-scale studies as they are limited to a single protein-protein docking simulation. EpiDOCK (Atanasova et al., 2013) is also one of the structure-based servers used for MHC binding peptide prediction using dock score-based QM.

Molecular dynamics (MD) simulation is a method for studying the movement of molecules and atoms in a realistic molecular system by utilising a force field to model intramolecular and intermolecular interactions at the atomic level. Using this technique, which numerically solves the time-dependent behaviour of a molecular system on a microscopic scale, the structure and conformational changes of proteins, as well as their thermodynamic properties, are examined in depth. It can also be used to investigate the dynamics and binding mode of novel peptide vaccines, interactions between peptide vaccines and the receptor binding groove, residue specificity and dissociation of MHC peptide-protein complexes, and interactions between the T-cell receptor and the MHC–peptide complex. AMBER (Pang, 2016), GROMACS (Kohnke, Kutzner & Grubmüller, 2020), CHARMM (Kim et al., 2020), and NAMD (Phillips et al., 2020) are some of the most used force fields and MD simulation programmes for calculating binding free energies.

**Figure 5:**
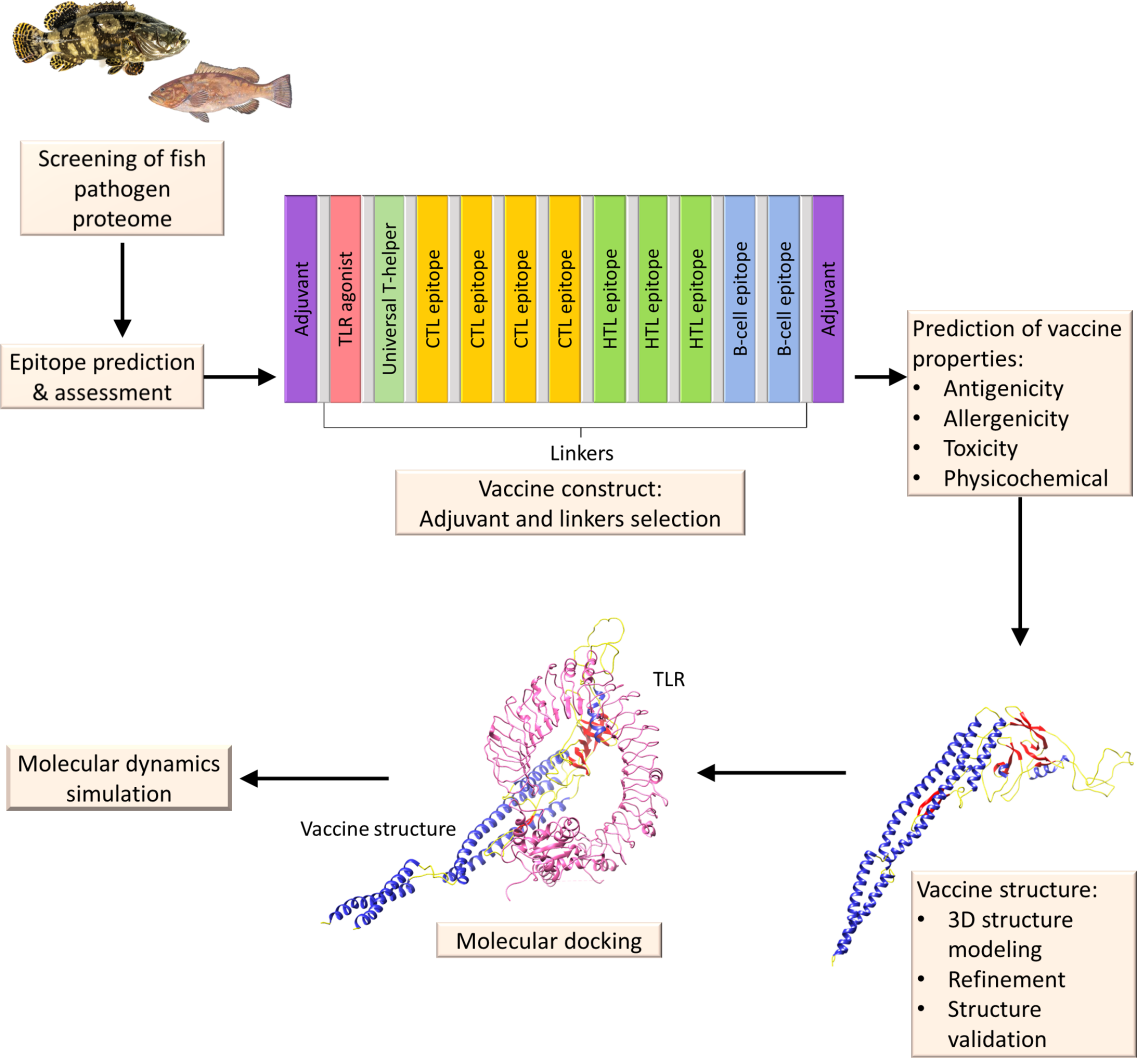
A diagrammatic description of the procedures involved in the *in silico* design of a multi-epitope vaccine for fish illnesses. Beginning with proteome retrieval and continuing through multi-epitope vaccine design and its validation by molecular docking and MD simulation.

The creation of these computational methods will facilitate the molecular analysis of peptide vaccines and receptor interactions, thereby facilitating the design and development of possible vaccinations against fish diseases. To design a multi-epitope subunit vaccine targeting the fish pathogen, the following immunoinformatics steps will be sequentially applied: (1) screening of the fish pathogen proteome, (2) B- and T-cell epitope prediction, (3) construction of vaccine by joining together the epitopes, linkers, and adjuvants, (4) vaccine properties prediction, (5) vaccine 3D structure modelling, (6) molecular docking with TLRs, and (7) MD simulations for stability (Fig. 5).

### Immune mechanism and immunoinformatics in fish disease

#### Immune mechanism prediction: TLR signalling pathways

To recognise an infection in an innate immune system, the toll-like receptor (TLR) is the receptor that has been researched extensively (Palti, 2011; Li et al., 2017). These receptors can also be referred to as a pattern recognition receptor (PRR) family that recognises, firstly, an external pathogen-associated molecular pattern (PAMP) derived from several types of microbial pathogen (Zhang & Liang, 2016) and, secondly, an internal damage-associated molecular pattern (DAMP) created by cells near death or tissues that have suffered damage (Yu & Feng, 2018). Different TLRs play an essential role in bridging the gap between innate and adaptive immunity by determining characteristics such as accurate identification and immune response to hazardous stimuli (El-Zayat, Sibaii & Mannaa, 2019).

Fish TLRs and the components involved in their signalling cascade share significant structural similarities with the mammalian TLR system. Despite this, the fish TLRs exhibit unique features and a wide range of diversity, which is likely due to their diverse evolutionary history and habitat. To date, thirteen TLR members have been discovered in mammals and each of which functions as a sensor for different PAMPs (Palti, 2011; Wang et al., 2021). In addition, TLRs are also present in fish where more than 21 TLRs have been reported so far (Liao et al., 2017). Fish have been shown to not possess TLR6, TLR10, TLR11, and TLR12. Furthermore, TLR4 which is absent in many species is found in some cyprinid fish, along with TLR5S, TLR18-TLR20, TLR23, and TLR25-TLR28, which are considered to be “fish-specific” TLRs (Rebl, Goldammer & Seyfert, 2010; Palti, 2011; Wang et al., 2015; He et al., 2019a).

**Figure 6:**
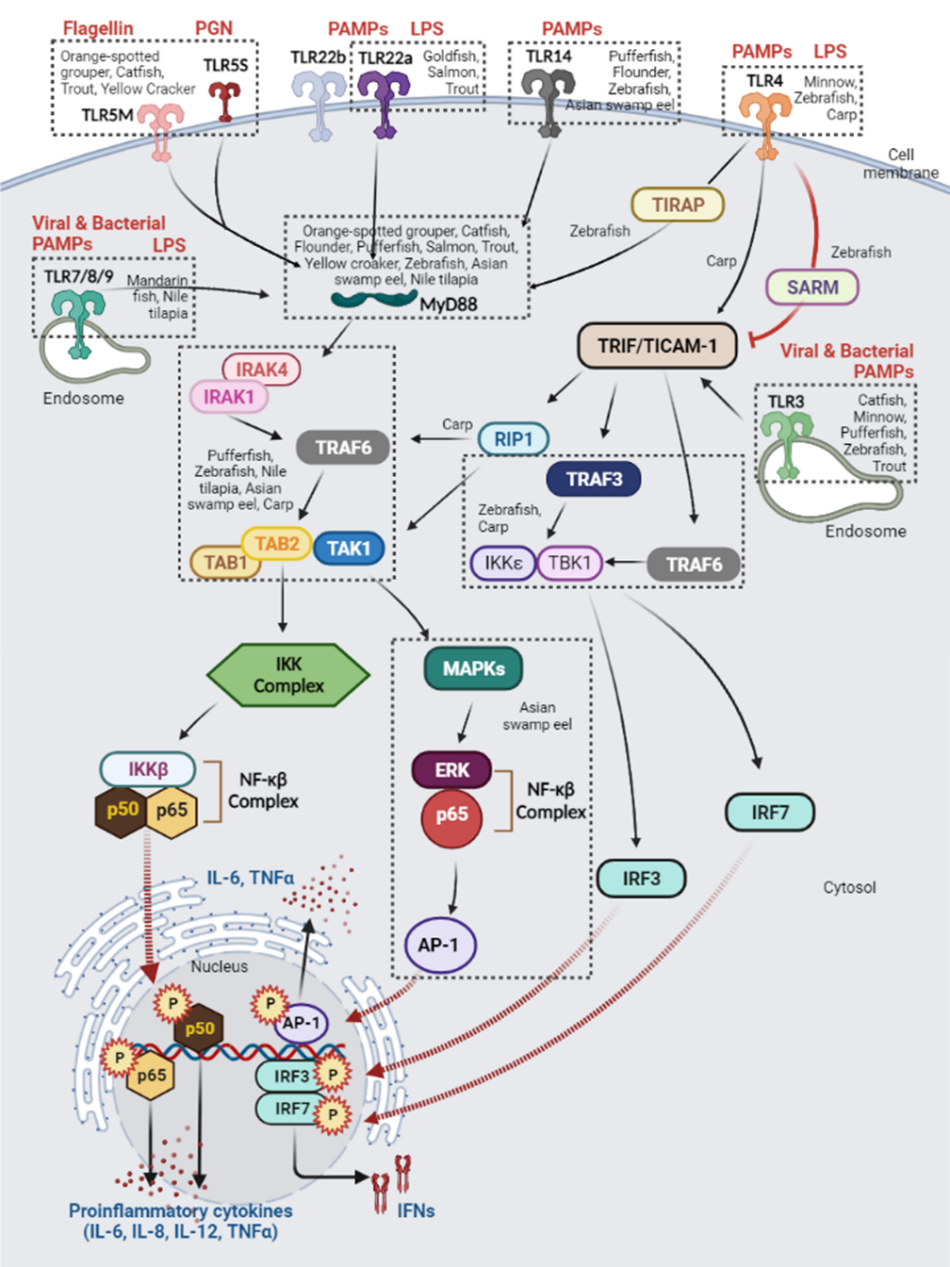
Schematic illustration of immune mechanisms activation in different fish species (boxed by dotted lines) through Toll-like receptor (TLR) signalling pathways. Modified from Rauta et al. (2014), Rebl, Goldammer & Seyfert (2010), and Zhang et al. (2014). Created with BioRender.com.

Recent studies on fish TLRs have concentrated on identifying individual TLR members in diverse teleost fish species such as TLR7 and TLR8 in Barbel chub (*Squaliobarbus curriculus*) (Jin et al., 2018), TLR21, TLR22, and TLR25 in Dabry’s sturgeon (*Acipenser dabryanus*) (Qi et al., 2018), TLR1-TLR3, TLR5, TLR7-TLR9. TLR13, TLR22, TLR25, and TLR26 in Walking catfish (*Clarias batrachus*) (Priyam et al., 2020). Furthermore, researchers have also examined what occurs when a pathogenic bacterium, virus, or ligand is used as a stimulator, with regard to expression profiles and signalling cascade genes (Jiang et al., 2020; Muduli et al., 2021; Wang et al., 2021). TLR ligands remain substantially unknown, especially in cartilaginous fish and lobe-finned fish (Nie et al., 2018; Smith, Rise & Christian, 2019). Several investigations detected TLR2, TLR3, TLR6, and TLR9 in the transcriptome data of the grey bamboo shark (*Chiloscyllium griseum*), (Anandhakumar et al., 2012; Krishnaswamy et al., 2014) while TLR3 was identified in the immunological response of the Nigerian spotted lungfish (*Protopterus dolloi*) (Tacchi, Misra & Salinas, 2013). Among all these TLRs, TLR4, TLR5, TLR9, and TLR14 are regarded as sensors of bacterial ligands while TLR3, TLR7, TLR8, and TLR22 are presumed viral ligands sensors (Rebl, Goldammer & Seyfert, 2010; Rauta et al., 2014; Zhang et al., 2014; Wang et al., 2021). Thus, in this review, the ligand specificity like PAMPs and signal pathways of fish TLRs are summarised in Fig. 6.

Theoretically, TLRs are activated when they recognise ligands which prompts the recruitment of adaptor molecules in the cytoplasm and the initiation of signalling cascades (Kenny & O’Neill, 2008; Luo et al., 2020). Both adaptors MyD88 (myeloid differentiation primary response 88) and TRIF (TIR-domain-containing adapter-inducing interferon- *β*) also known as TICAM-1 (TIR-containing adaptor molecule-1) are signals to downstream signalling pathways whereas the other one, TIRAP is primarily an adaptor for TLRs to connect to TRIF and MyD88, respectively (Troutman, Bazan & Pasare, 2012; DeFranco, 2016; Farooq et al., 2021) (Fig. 6). The activation of TLR signalling implicates at least two different pathways *i.e., via* the MyD88-dependent pathway which leads to the induction of various cytokines (IL-6, IL-8, IL-12, and TNF *α*) and MyD88-independent pathway which is associated with the induction of IFN and maturation of dendritic cells (Rauta et al., 2014; Farooq et al., 2021).

Through the MyD88-dependent pathway, MyD88 utilizes its death domain to interact with IRAK4 (IL-1 receptor-associated protein kinase 4) to form the MyD88-IRAK4 complex. This complex then phosphorylates IRAK2 or IRAK1 and recruits TRAF6 (tumour necrosis factor receptor-associated factor 6) *via* ubiquitination (Cao, Henzel & Gao, 1996; Fitzgerald & Kagan, 2020). Studies have reported that IRAK2 has been lost or not identified in fish (Zhang et al., 2014; Rebl et al., 2019), however, another study has stated that IRAK2 was found in the West Indian Ocean coelacanth (*Latimeria chalumnae*) genome (Li et al., 2018). Following ubiquitination, TRAF6 interacts and activates the TAB1/TAK1/TAB2 complex whereby TAB1 activates TAK1 (transforming growth factor-*β*-activated kinase 1) while TAB2 serves as an adaptor that connects TAK1 to TRAF6. TAK1 is then coupled to the IKK complex which leads to IKKßphosphorylation and the subsequent translocation of the NF-*κ*ßcomplex into the nucleus. NF-*κ*ßcomplex containing p50 and p65 combines with gene transcription to induce proinflammatory cytokines such as IL-6, IL-8, IL-12, and TNF*α* (Rebl, Goldammer & Seyfert, 2010; Rauta et al., 2014; Farooq et al., 2021).

TAK1 simultaneously phosphorylates MAPKs (mitogen-activated protein kinases) and induces the activation of AP-1 (activating protein-1) (Xu & Lei, 2021). For example, MaTLR14, a fish-specific TLR14 was identified in an Asian swamp eel (*Monopterus albus*) which increased TRAF6 expression and phosphorylation of ERK (extracellular signal-regulated kinase) and p65, thereby activating the NF-*κ*ßcomplex and AP-1. As a result, this phenomenon stimulated the production of proinflammatory cytokines such as IL-6 and TNF *α* (Zhang et al., 2014; Liu et al., 2022). Additionally, TLR14 was identified in the majority of fish orders, including pufferfish, zebrafish, flounder, golden pompano, and lamprey (Rebl, Goldammer & Seyfert, 2010; Wu et al., 2019; Sousa et al., 2022), and interestingly, it shared similar features to TLR6 and TLR10 of mammals, despite the absence of both TLRs in fish signalling cascades (Rauta et al., 2014; Liao et al., 2017).

Additionally, nearly every poikilothermic vertebrate (for instance, an amphibian or a fish) has exhibited TLR22, although it is not present in mammals. TLR22 has a vital part to the role in activating adaptive immunity and initiating innate immunity. In addition, the MyD88 adapter is required to initiate the signalling cascade (Paria et al., 2018; Ji et al., 2020; Gao et al., 2021a). Similarly, to TLR4, TLR5 activates the MyD88-dependent pathway and consists of two distinct forms, the membrane TLR5 (TLR5M) and soluble TLR5 (TLR5S). Together, they detected the bacterial flagellin, despite having opposing roles in stimulating the signalling cascade. The flagellin induced the basal activation of NF-*κ*ß*via* TLR5M, causing the activation of TLR5S expression in the liver. TLR5S is efficient in binding to circulating flagellin and transporting the latter to the membrane TLR5 factor, which amplifies the signalling of danger in positive loop feedback pathways (Rebl, Goldammer & Seyfert, 2010; Zhang et al., 2014; Jiang et al., 2017; Jiang et al., 2020; He et al., 2019b). This phenomenon is observed in the stimulation of *Vibrio parahaemolyticus* flagellin in orange-spotted grouper (*Epinephelus coioides*) (Bai et al., 2017; He et al., 2019a) and large yellow croaker (*Larimichthys crocea*) (Jiang et al., 2020) as well as *Yersinia ruckeri* stimulation in rainbow trout (*Oncorhynchus mykiss*) (Wangkahart, Secombes & Wang, 2019) and channel catfish (*Ictalurus punctatus*) (Jiang et al., 2017).

In contrast to the three TLR family members TLR7, TLR8, and TLR9, bacterial and viral PAMPs do not induce their cell signalling cascades in endosomes after being activated by lipopolysaccharides (LPS). The MyD88-dependent pathway considerably elevated their expression levels in all investigated tissues of Nile tilapia (*Oreochromis niloticus*) and mandarin fish (*Siniperca chuatsi*) (Gao et al., 2021b; Wang et al., 2021). In addition, the foregoing results indicate that the MyD88-dependent pathway in fish is comparable to all TLRs except TLR3 (Rauta et al., 2014; Zhang et al., 2014; Fitzgerald & Kagan, 2020).

In addition, TLR4 in zebrafish (Danio rerio) uses alternative adaptor proteins such as TIRAP to recruit MyD88 to activate IRAK following the induction of LPS and PAMPs, as depicted in Fig. 6 (Rauta et al., 2014; Li et al., 2017; Loes et al., 2021). In addition to TIRAP, additional adaptor molecules, such as SARM (sterile alpha and HEAT/Armadillo motif-containing protein), have been identified in zebrafish. SARM is the only adaptor protein that inhibits TLR signalling by interacting with TRIF (red arrow in Fig. 6). Its expression inhibited the function of TRIF *via* the TLR3 and TLR4 pathways, whereas its silencing had the opposite effect (Peng et al., 2010; Kanwal et al., 2014; Loring & Thompson, 2020; Luo et al., 2020).

The recruitment of TRIF to TLR4 and TLR3 occurs, thereby promoting an alternative avenue which results in IRF3, NF-ß, AP-1, MAPKs, and IRF3 (interferon regulatory factor 3) being activated to produce proinflammatory cytokines and/or IFN1 (type I interferon) (Kawasaki & Kawai, 2014; Hu et al., 2015; Nie et al., 2018). In addition, NF-*κ*ßin the TRIF-dependent pathway can be activated *via* the recruitment of RIP1 and TRAF6 *via* the C-terminal RHIM domain and the TRAF6 binding motif, respectively (Zhang et al., 2020; Liu et al., 2021). Intriguingly, the activation of NF-*κ*ßin carp TRIF was consistent with the findings in large yellow cracker, orange-spotted grouper, and zebrafish, indicating that fish TRIF in the NF-*κ*ß-mediating signalling cascade has a conserved function (Zhang et al., 2020; Liu et al., 2021; Zou et al., 2021).

Unlike TLR4, TLR3 interacts directly with TICAM-1 (also known as TRIF) in the MyD88-independent pathway. The TLR3-TICAM-1 signalling pathway is one of the most important immune responses to RNA virus infection (Rebl, Goldammer & Seyfert, 2010; Nie et al., 2018; Geng et al., 2021). As a result, it induces IFN1 production by activating IFN3 and IFN7 *via* an interaction with TRAF3 and TRAF6 (Li et al., 2017; Geng et al., 2021). As shown in Fig. 6. TLR3 has been identified in numerous fish species, such as channel catfish (*Ictalurus punctatus*), rare minnow (*Gobiocypris rarus*), pufferfish (*Takifugu rubripes*), zebrafish (*Danio rerio*), and rainbow trout (*Oncorhynchus mykiss*).

To conclude, the TLR cascade involves the recruitment of components whose functions resemble each other or are identical in every species of vertebrate apart from fish, whose attributes are unique. More downstream components of this signalling cascade must be studied in bony fish (teleost fish, cartilaginous fish and lungfish) to elucidate the functional similarities and divergences of TLR signalling in a fish with bones or a mammal. The importance of this is due to TLR working to connect innate and adaptive immunity, which should facilitate the understanding of how a fish vaccine functions.

#### Multi-epitope vaccine and treatment

Compared to conventional vaccinology, epitope-based chimeric (subunit) vaccines using an immunoinformatics approach offer many advantages such as not requiring microbial culturing, being less expensive to develop, taking less time to produce, outperforming numerous wet-lab experiments, and being specific and stable because they do not contain the entire organism (Kar et al., 2020; Naz et al., 2020; Bukhari et al., 2022). Due to the occurrence of MHC variants, an epitope-based vaccine targeting limited MHC alleles typically does not have the desired or equivalent effect on the fish population. Consequently, very promiscuous epitopes can simultaneously bind different alleles, allowing for the immunological response sought in a diverse fish population (Wegner, 2008; Patronov & Doytchinova, 2013; Radwan et al., 2020; Šimková et al., 2021). This approach, termed a multi-epitope vaccine, consists of a set of peptides that overlap, and it induces an immune response according to a short immunogenic sequence (Zhang et al., 2012).

A multi-epitope vaccine also uses certain design principles, such as TH, B-cell, and CTL epitopes, which can cause strong cellular and humoral immunity at the same time; many MHC-restricted epitopes, which a TCR from a different subset of T-cells can recognize; and many epitopes from different forms of antigens, which increases the number of bacteria and viruses that can be targetedFurthermore, multi-epitope vaccines involve the introduction of an adjuvant-capable element able to boost immunogenicity and offer durable immune responses. Moreover, undesirable elements that might cause an abnormal immune response or a detrimental side effect can be eliminated (Saadi, Karkhah & Nouri, 2017; Zhang, 2018; Sami et al., 2021; Sanches et al., 2021).

Recently, an *in-silico* method was able to accurately predict epitopes and multi-epitopes with remarkable responsiveness against *Streptococcus agalactiae*, *Streptococcus iniae*, *Edwardsiella tarda*, and *Flavobacterium columnarie* individually (Forouharmehr et al., 2022b; Islam, Mou & Sanjida, 2022). Pathogenic bacteria such as *Streptococcus agalactiae* have caused streptococcus’s disease in tilapia aquaculture (Toranzo, Magariños & Romalde, 2005; Su et al., 2016; Sirimanapong et al., 2018). Tilapia species such as Nile tilapia, *Oreochromi* s *niloticus* Linn. that are infected with *Streptococcus agalactiae* typically exhibit various symptoms such as dark skin pigment, dermal haemorrhages, hyperaemic gills, eye lesions, erratic swimming, spinal curvature, and diffuse epithelial tissue proliferation (Geng et al., 2012; Su et al., 2016; Yi et al., 2019). In addition, the currently available vaccines have limits in protecting fish against catastrophic death when infected with different strains of *Streptococci* sp. (Pereira et al., 2013; Mishra et al., 2018; Pumchan et al., 2020). Utilizing immunoinformatics, a strategy to design a multi-epitope vaccine was implemented to address this problem. As a result, two of five antigenic proteins (45F2 and 42E2) were predicted as the best candidates for constructing a multi-epitope vaccine and were subsequently shown to successfully protect against streptococcus’s disease in tilapia (Pumchan et al., 2020).

Similarly, the most recent study reported by Forouharmehr et al. (2022b) described the effectiveness of multi-epitope vaccinations that employ a number of immunogenic proteins. The prediction of the epitopes involved six immunogenic Streptococcus iniae proteins: GAPDH, MtsB, ENO, Sip11, FBA, and SCPI. In this context, the most suitable multi-epitope vaccine was constructed by foreseeing various epitopes, such as the B-cell, T-cell, and IFN *γ* epitopes of the immunogenic proteins and interleukin-8 (IL-8). An analysis was also conducted of the vaccine’s antigenicity, physicochemical attributes, and secondary and tertiary structural forms, as well as different aspects deemed vital in the vaccine’s development. Additionally, this study revealed that the developed vaccine’s IL-8 domain had the highest level of binding affinity when docking with its receptor, and this was adapted with success so that it could be expressed in *Escherichia coli*. As a result, a stable vaccine with an antigenicity score of 0.936 and a 45-kDa molecular weight has been developed. This multi-epitope vaccine looks to be an effective candidate for preventing *Streptococcus iniae* infections in fish.

Additional pathogenic bacteria such as *Edwardsiella tarda* and *Flavobacterium columnare* also cause Edwardsiellosis and Columnaris diseases in the majority of fish species resulting in a high mortality rate among distinct populations of fish of varying ages (Sudheesh et al., 2012; Declercq et al., 2013; Hirai et al., 2015; Zhou et al., 2018). The *E. tarda*-infected fish displayed symptoms such as abnormal swimming, spiral movement, and floating near the water’s surface. This virulent intracellular pathogen poses serious threats, particularly in the farming of catfish, flounder, turbot, yellowtail, and tilapia species (Park, Aoki & Jung, 2012; Mahendran et al., 2016a; Miniero Davies et al., 2018). In the meantime, columnaris disease caused by *F. columnare* mostly affects fish species such as goldfish, channel catfish, eels, tilapia, carp, perch, and salmonids. This virulent bacterium is found in the individual gill filaments and causes yellowish-brown lesions on the gills, fin, and skin (Arias et al., 2012; Zhu et al., 2012; Mahendran et al., 2016a; Mitiku, 2018). The need for novel vaccines against edwardsiellosis and columnaris illnesses has increased as a result of these concerns. Various antibiotics, including colistin, rifampin, oxacillin, and penicillin, have been used to control edwardsiellosis (Mahendran et al., 2016a), whilst quinolones and tetracyclines are used against columnaris infection (Mitiku, 2018). In spite of this, the extensive use of antibiotics results in the evolution of various drug resistances and has caused the enormous deaths of farmed and wild fishes owing to bacterial infection (Kumar et al., 2012; Beck et al., 2015; Abd El-Tawab et al., 2020; Preena, Dharmaratnam & Swaminathan, 2022).

Although vaccines for columnaris treatment are now available, there is a risk of reversion in certain cases of live attenuated vaccines. Similarly, the possibility of a monovalent vaccine to protect all the susceptible fish hosts from *Edwardsiella* sp. is impossible. This is because the bacterium possesses different host-based genotypes such as serological, genetic, and antigenic heterogenous (Park, Aoki & Jung, 2012; Mahendran et al., 2016a; Buján, Toranzo & Magariños, 2018; Bothammal et al., 2021). Therefore, this risk of reversion could be prevented by predicting the B-cell and T-cell epitopes in peptide sequences and then developing an effective multi-epitopes vaccination using an immunoinformatics technique. The docked structure of peptide-MHC I complexes has been successfully modelled using two and five CTL epitopes of outer membrane proteins (OMPs) from *E. tarda* and *F. columnare*, respectively, according to a prior study described by Mahendran et al. (2016a). Their interactions were studied using immunoinformatics tools. In addition, infection by other bacterial strains of *Edwardsiella* sp. in fish has also been reported recently. A multi-epitope chimeric protein, EiCh is composed of eleven B-cell epitopes and seven MHC II epitopes that were successfully constructed and expressed in *E. coli* BL-21 (DE3). As a consequence, 49.32-kDa recombinant EiCh protein induced a potent antibody response against *E. ictaluri* in Nile tilapia and striped catfish. This finding indicates that the immunoinformatics strategy for vaccine formulation studied in this study is essential for treating *Edwardsiella* sp. infections in fish species (Machimbirike et al., 2022b).

In addition to bacterial illnesses, viral infections have a negative impact on aquaculture productivity. For instance, viral encephalopathy and retinopathy are caused by the nervous necrosis virus (NNV), leading to extensive death rates in commercially farmed species of fish that can exceed 100% (Hazreen-Nita et al., 2019; Kaushik, 2020; Michel-Todó et al., 2020). In this regard, an *in-silico* method was utilised to design an epitope-based vaccine to protect grouper and sea bass fish species from NNV infections. Six antigenic epitopes were selected from a pool of one thousand and conjugated with adjuvant and linker peptides. As a result, the model of an engineered epitope-based vaccine showed good binding to toll-like receptor-5 (TLR5), a crucial elicitor of the immune response. This prediction would be useful prior to cloning and purifying the NNV 248-specific protein (Joshi et al., 2021).

In addition to affecting marine and shellfish species, the marine birnavirus (MABV) outbreak has a significant economic impact on aquaculture production (Mancheva et al., 2021; Fu et al., 2022). In shorter periods in standard culture conditions, MABV is the most pathogenic virus to have resulted in complete mortality, hence the limitations on being able to prevent this virus (Crane & Hyatt, 2011; Diggles, 2016; Chen et al., 2019; Islam, Mou & Sanjida, 2022). Thus, an immuno-informatics method was employed to construct an epitope-based vaccine against MABV by recognising the most pathogenic and antigenic proteins of MABV; RNA-dependent RNA polymerase (RdRp), polyprotein (PP), and major capsid protein VP2 (MCPVP2) of MABV. For all the proteins, the leading three CTL epitopes with the most appropriate adjuvants and linkers to ensure non-allergenity, immunogenity, and better solubility were anticipated so that the multi-epitope birnavirus (MEBV) could be designed. Using E. coli K12 as a model, codon optimisation was conducted to improve the translational efficiency of the vaccine design. The codon was ultimately modified, and *in silico* cloning using the E. coli K12 expression host, pET28a (+) vector, was effective. This potential peptide vaccine might be an effective MABV preventive strategy (Islam, Mou & Sanjida, 2022).

Using immunoinformatics methodologies prior to conducting wet-lab trials, the design of multi-epitope vaccines is a successful method for combating the majority of infectious diseases, it may be concluded. This demonstrates that this method is one of the most effective ways to manage bacterial and viral infections in commercial fish species.

#### Fish vaccine design based on immunoinformatics approach: promising strategies

As we consider the future of immunoinformatics in fish vaccine design, it will be of the utmost importance to cultivate collaboration and knowledge sharing within this field. Several promising strategies can be employed to achieve this goal:

i. The establishment of open-access platforms for researchers to share their data, methodologies, and immunoinformatics tools is essential to fostering knowledge exchange. These platforms could include preprint servers for early dissemination of research findings, data repositories for sharing raw datasets, and open-source bioinformatics software repositories. The collective exchange of information could accelerate progress in this field by minimising duplication of effort and allowing scientists to iterate on previous work.
ii. Promoting international collaborations could be instrumental in combining diverse skills, resources, and points of view. Networks that facilitate collaboration between immunologists, bioinformaticians, and aquaculture specialists, among others, would expedite the integration of diverse ideas and methodologies, thereby fostering innovation in the field.
iii. Introducing capacity-building programmes and workshops in immunoinformatics and related disciplines would equip researchers with the necessary knowledge and skills, particularly those in regions with limited resources. This would enable more scientists to contribute to the field and cultivate a research community that is more globally representative.
iv. Policymakers can provide impetus by creating policies that promote data exchange, collaboration, and research in this field. Furthermore, the allocation of funds specifically for aquaculture immunoinformatics research would stimulate activity and promote innovation in this field.
v. Promoting collaboration between public research institutions and private industry could pool resources and expertise, thereby accelerating the translation of research findings into practical applications, such as novel fish vaccines.
vi. Ethics and Governance: As collaborations expand and research becomes more data-driven, it is crucial to establish robust ethical and governance frameworks. These protocols should include data privacy, intellectual property rights, and equitable benefit-sharing in order to ensure the ethical and efficient execution of collaborations.

Attention should be given to the potential of immunoinformatics and multi-epitope vaccine design to improve vaccine manufacturing capacity, particularly in low- and middle-income countries (LMICs). These computational approaches, which rely on accessible and affordable digital resources rather than costly laboratory infrastructure, have the potential to revolutionise vaccine development in environments with limited resources. Immunoinformatics can reduce the cost of vaccine development, a crucial factor for LMICs. It facilitates the antigen discovery procedure by enabling researchers to predict antigenic components of a pathogen that are likely to elicit a robust immune response using computational methods. This avoids the costly and time-consuming process of empirical experimentation, making the development of vaccines more affordable for institutions with limited resources. Moreover, multi-epitope vaccines, which contain sequences from multiple epitopes, provide additional benefits. They are typically manufactured synthetically, allowing LMICs to potentially produce their own vaccines instead of relying on imports. This factor could result in substantial cost savings and increase local biotechnology expertise.

In addition, the reduced cold chain requirements of multi-epitope vaccines, as a result of their increased thermal stability, could alleviate the logistical challenges associated with vaccine distribution in LMICs. In these regions, where maintaining the necessary low-temperature conditions is often challenging, overcoming cold chain constraints is a pressing necessity. Lastly, the adaptability of multi-epitope vaccine design can pave the way for the development of custom vaccines. These could be modified to target specific pathogen strains prevalent in certain geographic regions, enabling more effective and targeted immunisation strategies. Implementing these strategies could considerably increase collaboration and information exchange in the field of immunoinformatics-based fish vaccine design. By doing so, we can foster a global, collaborative research community with the common objective of developing more effective and sustainable aquaculture health management solutions.

## Conclusions

Vaccination is fundamental to the sustainable management of aquaculture, playing a crucial role in preventing disease outbreaks and the overuse of antibiotics in fish. However, research and development on vaccines for aquatic animals are still in their infancy, highlighting the pressing need for enhanced strategies. The development of species-specific vaccines requires a comprehensive understanding of fish immune systems, including B-cells, T-cells, MHC molecules, and TLR signalling pathways; however, traditional approaches are often costly and time-consuming. Immunoinformatics is a prospective alternative for more effective vaccine design, capable of addressing the complexities of emerging and re-emerging diseases, antigenic diversity, and personalised immunisation requirements. This strategy employs high-performance tools for identifying multi-epitope vaccines, thereby providing a platform for examining variations in immune adaptations among fish species. However, there are gaps and limitations in the discipline.

Despite the promise of immunoinformatics, the accuracy of these predictions is inextricably linked to the precision of the data and the sophistication of the algorithms employed. Inaccuracies in high-throughput sequence data highlight the need for robust data cleansing methods and verification protocols; without them, subsequent analyses may be erroneous. Future research should concentrate on refining the computational techniques used in immunoinformatics and developing better methods for integrating these techniques with conventional laboratory testing. This will not only result in a more precise vaccine design based on epitopes, but it will also promote a targeted, cost-effective, and safer approach to fish vaccination. Moreover, the establishment of a comprehensive database of fish immune responses at the individual, species, and population levels could provide valuable insights for vaccine design and delivery strategies. Although immunoinformatics is a promising instrument for vaccine development, it must be continuously refined and validated. In order to push the boundaries of aquatic animal health management in the coming years, it will be essential to adopt this technology and simultaneously address its gaps and limitations.

Considering the undeniable fact that the increase in immunoinformatics studies following COVID-19 has broadened our understanding and presented numerous potential vaccine candidates, the true value of these findings cannot be confirmed without laboratory validation. Computational analysis and *in silico* modelling are essential instruments, but they are only the first step in a multi-step process leading to the development of a viable vaccine. The majority of published immunoinformatics studies are indeed predominantly computational, and their contribution to actual vaccine development can be limited in the absence of experimental validation. The prediction of potential antigens or epitopes is based on our current knowledge of protein structure and immune response, both of which are still active research areas. In addition, immunoinformatics tools are imperfect and frequently operate based on assumptions that may not always be true. Therefore, experimental data must always validate bioinformatics predictions.

Determining the protective efficacy of identified vaccine candidates in aquaculture is difficult due to the complexity of immune responses in aquatic organisms and the difference between their immune systems and those of mammals. Important is experimental validation in the form of laboratory and field evaluations. These include, but are not limited to, epitope mapping, evaluating for immune response in cell cultures or fish, and observing the progression of disease resistance in vaccinated populations. Although the redundancy of theoretical vaccine papers can be a cause for concern, it is essential to remember that these studies still contribute to our collective knowledge and may serve as the foundation for future experimental research. Increasing accessibility to computational tools and techniques has democratised scientific research, allowing more scientists to contribute their findings. As these findings are tested and validated in the laboratory, we will be able to enhance the predictive power of immunoinformatics by refining our tools and models.

To ensure that immunoinformatics research effectively contributes to the development of essential vaccines, it is crucial to encourage the laboratory application of these computational findings. This can be accomplished by encouraging collaboration between computational and experimental biologists, promoting funding for validation studies, and emphasising the publication of studies that include both computational and experimental components. In conclusion, although the influx of theoretical vaccine papers provides a plethora of potential vaccine candidates, it is essential to take these findings to the next level by conducting the necessary experimental validations to advance vaccine development in the aquaculture field.

## Supplemental Information

10.7717/peerj.16419/supp-1 Supplemental Information 1 A summary of tools useful in immunoinformatics vaccine design for fish diseases

## Additional Information and Declarations

### Data Availability

The following information was supplied regarding data availability: This is a literature review.

## References

[ref-1] Abd El-Tawab A, El-Hofy F, EL-Gamal R, Awad S, El-Mougy E, Mohamed S (2020). Phenotypic and genotypic characterization of antibiotic resistant strains of Flavobacterium columnare isolated from Oreochromis niloticus (Nile tilapia). Benha Veterinary Medical Journal.

[ref-2] Abdelhamed H, Lawrence ML, Karsi A (2018). Development and characterization of a novel live attenuated vaccine against enteric septicemia of catfish. Frontiers in Microbiology.

[ref-3] Abdelrahman HA, Hemstreet WG, Roy LA, Hanson TR, Beck BH, Kelly AM (2023). Epidemiology and economic impact of disease-related losses on commercial catfish farms: a seven-year case study from Alabama, USA. Aquaculture.

[ref-4] Abinaya RV, Viswanathan P, Hasija Y (2021). Chapter 2 - Biotechnology-based therapeutics. Translational biotechnology.

[ref-5] Abudula T, Bhatt K, Eggermont LJ, O’Hare N, Memic A, Bencherif SA (2020). Supramolecular self-assembled peptide-based vaccines: current state and future perspectives. Frontiers in Chemistry.

[ref-6] Adams A (2019). Progress, challenges and opportunities in fish vaccine development. Fish and Shellfish Immunology.

[ref-7] Alekseenko A, Ignatov M, Jones G, Sabitova M, Kozakov D (2020). Protein—protein and protein—peptide docking with ClusPro server. Methods in Molecular Biology.

[ref-8] Alogheli H, Olanders G, Schaal W, Brandt P, Karlén A (2017). Docking of macrocycles: comparing rigid and flexible docking in glide. Journal of Chemical Information and Modeling.

[ref-9] Anandhakumar C, Lavanya V, Pradheepa G, Tirumurugaan KG, Dhinakar Raj G, Raja A, Pazhanivel N, Balachandran C (2012). Expression profile of toll-like receptor 2 mRNA in selected tissues of shark (Chiloscyllium sp.). Fish and Shellfish Immunology.

[ref-10] Ángeles Esteban M (2012). An overview of the immunological defenses in fish skin. International Scholarly Research Network Immunology.

[ref-11] Antunes DA, Devaurs D, Moll M, Lizée G, Kavraki LE (2018). General prediction of peptide-MHC binding modes using incremental docking: a proof of concept. Scientific Reports.

[ref-12] Arias CR, Cai W, Peatman E, Bullard SA (2012). Catfish hybrid Ictalurus punctatus x I. furcatus exhibits higher resistance to columnaris disease than the parental species. Diseases of Aquatic Organisms.

[ref-13] Assefa A, Abunna F (2018). Maintenance of fish health in aquaculture: review of epidemiological approaches for prevention and control of infectious disease of fish. Veterinary Medicine International.

[ref-14] Atanasova M, Patronov A, Dimitrov I, Flower DR, Doytchinova I (2013). EpiDOCK: a molecular docking-based tool for MHC class II binding prediction. Protein Engineering, Design and Selection.

[ref-15] Bai J, Li Y, Deng Y, Huang Y, He S, Dai J, Zhao S-Z, Dan X-M, Luo X-C (2017). Molecular identification and expression analysis of TLR5M and TLR5S from orange- spotted grouper (Epinepheluscoioides). Fish and Shellfish Immunology.

[ref-16] Beck BH, Barnett LM, Farmer BD, Peatman E, Carter D (2015). Kaolinitic clay protects against Flavobacterium columnare infection in channel catfish Ictalurus punctatus (Rafinesque). Journal of Fish Diseases.

[ref-17] Bedekar MK, Kole S, Tripathi G, Malik YS, Barh D, Azevedo V, Paul Khurana SM (2020). Chapter 17 - Biotechnological approaches to fish vaccine. Genomics and biotechnological advances in veterinary, poultry, and fisheries.

[ref-18] Bi B, Yuan Y, Jia D, Jiang W, Yan H, Yuan G, Gao Y (2023). Identification and pathogenicity of emerging fish pathogen Acinetobacter johnsonii from a disease outbreak in rainbow trout (Oncorhynchus mykiss). Aquaculture Research.

[ref-19] Biller-Takahashi JD, Urbinati EC (2014). Fish immunology. The modification and manipulation of the innate immune system: Brazilian studies. Anais Da Academia Brasileira de Ciencias.

[ref-20] Bøgwald J, Dalmo RA (2019). Review on immersion vaccines for fish: an update 2019. Microorganisms.

[ref-21] Boehm T, Iwanami N, Hess I (2012). Evolution of the immune system in the lower vertebrates.

[ref-22] Bothammal P, Ganesh M, Vigneshwaran V, Anbarasu K, Ponmurugan K, Al-Dhabi NA, Natarajaseenivasan K (2021). Construction of genomic library and screening of Edwardsiella tarda immunogenic proteins for their protective efficacy against edwardsiellosis. Frontiers in Immunology.

[ref-23] Bouazzaoui A, Abdellatif AAH, Al-Allaf FA, Bogari NM, Al-Dehlawi S, Qari SH (2021). Strategies for vaccination: conventional vaccine approaches versus new-generation strategies in combination with adjuvants. Pharmaceutics.

[ref-24] Brisse M, Vrba SM, Kirk N, Liang Y, Ly H (2020). Emerging concepts and technologies in vaccine development. Frontiers in Immunology.

[ref-25] Buchmann K (2014). Evolution of innate immunity: clues from invertebrates via fish to mammals. Frontiers in Immunology.

[ref-26] Buján N, Toranzo AE, Magariños B (2018). Edwardsiella piscicida: a significant bacterial pathogen of cultured fish. Diseases of Aquatic Organisms.

[ref-27] Bukhari SNH, Jain A, Haq E, Mehbodniya A, Webber J (2022). Machine learning techniques for the prediction of B-Cell and T-cell epitopes as potential vaccine targets with a specific focus on SARS-CoV-2 pathogen: a Review. Pathogens.

[ref-28] Cai W, Arias CR (2021). Deciphering the molecular basis for attenuation of Flavobacterium columnare strain fc1723 used as modified live vaccine against columnaris disease. Vaccine.

[ref-29] Cao Z, Henzel WJ, Gao X (1996). IRAK: a kinase associated with the interleukin-1 receptor. Science.

[ref-30] Castro KM, Scheck A, Xiao S, Correia BE (2022). Computational design of vaccine immunogens. Current Opinion in Biotechnology.

[ref-31] Chatanaka MK, Ulndreaj A, Sohaei D, Prassas I (2022). Immunoinformatics: pushing the boundaries of immunology research and medicine. Immunoinformatics.

[ref-32] Chaudhary K, Nagpal G, Dhanda SK, Raghava GPS (2016). Prediction of immunomodulatory potential of an RNA sequence for designing non-toxic siRNAs and RNA-based vaccine adjuvants. Scientific Reports.

[ref-33] Chen J, Toh X, Ong J, Wang Y, Teo XH, Lee B, Wong PS, Khor D, Chong SM, Chee D, Wee A, Wang Y, Ng MK, Tan BH, Huangfu T (2019). Detection and characterization of a novel marine birnavirus isolated from Asian seabass in Singapore. Virology Journal.

[ref-34] Collins C, Lorenzen N, Collet B (2019). DNA vaccination for finfish aquaculture. Fish and Shellfish Immunology.

[ref-35] Côté-Gravel J, Brouillette E, Malouin F (2019). Vaccination with a live-attenuated small-colony variant improves the humoral and cell-mediated responses against Staphylococcus aureus. PLOS ONE.

[ref-36] Crane M, Hyatt A (2011). Viruses of fish: an overview of significant pathogens. Viruses.

[ref-37] Dadar M, Dhama K, Vakharia VN, Hoseinifar SH, Karthik K, Tiwari R, Khandia R, Munjal A, Salgado-Miranda C, Joshi SK (2017). Advances in aquaculture vaccines against fish pathogens: global status and current trends. Reviews in Fisheries Science and Aquaculture.

[ref-38] D’Amico C, Fontana F, Cheng R, Santos HA (2021). Development of vaccine formulations: past, present, and future. Drug Delivery and Translational Research.

[ref-39] Damodharan K, Arumugam GS, Ganesan S, Doble M, Thennarasu S (2021). A comprehensive overview of vaccines developed for pandemic viral pathogens over the past two decades including those in clinical trials for the current novel SARS-CoV-2. RSC Advances.

[ref-40] Declercq AM, Haesebrouck F, Van Den Broeck W, Bossier P, Decostere A (2013). Columnaris disease in fish: a review with emphasis on bacterium-host interactions. Veterinary Research.

[ref-41] DeFranco AL, Ratcliffe MJH (2016). Signaling pathways downstream of TLRs and IL-1 family receptors. Encyclopedia of immunobiology.

[ref-42] Delghandi MR, El-Matbouli M, Menanteau-Ledouble S (2020). Renibacterium salmoninarum—the causative agent of bacterial kidney disease in salmonid fish. Pathogens.

[ref-43] Dhar AK, Manna SK, Thomas Allnutt FC (2014). Viral vaccines for farmed finfish. Indian Journal of Virology.

[ref-44] Dias WO, Van Der Ark AAJ, Sakauchi MA, Kubrusly FS, Prestes AFRO, Borges MM, Furuyama N, Horton DSPQ, Quintilio W, Antoniazi M, Kuipers B, Van Der Zeijst BAM, Raw I (2013). An improved whole cell pertussis vaccine with reduced content of endotoxin. Human Vaccines and Immunotherapeutics.

[ref-45] Diggles B (2016). Updated disease risk assessment—relocation of salmon farms in Marlborough Sounds, New Zealand. DigsFish services report no. DF 16-01 for the ministry for primary industries.

[ref-46] Dimitrov I, Bangov I, Flower DR, Doytchinova I (2014). AllerTOP v.2—a server for in silico prediction of allergens. Journal of Molecular Modeling.

[ref-47] Divya MO, Devi AK (2023). Artificial intelligent fish abundance detector model for preserving environmental stability amid aquatic sustenance and fishermen. Journal of Survey in Fisheries Sciences.

[ref-48] Doytchinova IA, Guan P, Flower DR (2006). EpiJen: a server for multistep T cell epitope prediction. BMC Bioinformatics.

[ref-49] Du Z, Su H, Wang W, Ye L, Wei H, Peng Z, Anishchenko I, Baker D, Yang J (2021). The trRosetta server for fast and accurate protein structure prediction. Nature Protocols.

[ref-50] Dudek NL, Perlmutter P, Aguilar M-I, Croft NP, Purcell AW (2010). Epitope discovery and their use in peptide based vaccines. Current Pharmaceutical Design.

[ref-51] Eberhardt J, Santos-Martins D, Tillack AF, Forli S (2021). AutoDock Vina 1.2.0: new docking methods, expanded force field, and python bindings. Journal of Chemical Information and Modeling.

[ref-52] El-Manzalawy Y, Dobbs D, Honavar V (2008). Predicting linear B-cell epitopes using string kernels. Journal of Molecular Recognition.

[ref-53] El-Manzalawy Y, Honavar V (2010). Recent advances in B-cell epitope prediction methods. Immunome Research.

[ref-54] El-Zayat SR, Sibaii H, Mannaa FA (2019). Toll-like receptors activation, signaling, and targeting: an overview. Bulletin of the National Research Centre.

[ref-55] Embregts CWE, Forlenza M (2016). Oral vaccination of fish: lessons from humans and veterinary species. Developmental and Comparative Immunology.

[ref-56] Evensen Ø (2016). Immunization strategies against Piscirickettsia salmonis infections: review of vaccination approaches and modalities and their associated immune response profiles. Frontiers in Immunology.

[ref-57] Farooq M, Batool M, Kim MS, Choi S (2021). Toll-like receptors as a therapeutic target in the era of immunotherapies. Frontiers in Cell and Developmental Biology.

[ref-58] Feng P, Zeng J, Ma J (2021). Predicting MHC-peptide binding affinity by differential boundary tree. Bioinformatics.

[ref-59] Fernández Sánchez JL, Le Breton A, Brun E, Vendramin N, Spiliopoulos G, Furones D, Basurco B (2022). Assessing the economic impact of diseases in Mediterranean grow-out farms culturing European sea bass. Aquaculture.

[ref-60] Fitzgerald KA, Kagan JC (2020). Toll-like receptors and the control of immunity. Cell.

[ref-61] Fleri W, Paul S, Dhanda SK, Mahajan S, Xu X, Peters B, Sette A (2017). The immune epitope database and analysis resource in epitope discovery and synthetic vaccine design. Frontiers in Immunology.

[ref-62] Flower DR, Doytchinova I, Zaharieva N, Dimitrov I (2017). Immunogenicity prediction by VaxiJen: a ten year overview. Journal of Proteomics & Bioinformatics.

[ref-63] Forouharmehr A, Banan A, Mousavi SM, Jaydari A (2022a). Development of a novel multi-epitope vaccine candidate against Streptococcus iniae infection in fish: an immunoinformatics study. Archives of Razi Institute.

[ref-64] Forouharmehr A, Banan A, Mousavi SM, Jaydari A (2022b). Development of a novel multi-epitope vaccine candidate against Streptococcus iniae infection in fish: an immunoinformatics study. Archives of Razi Institute.

[ref-65] Fu X, Luo M, Zheng G, Liang H, Liu L, Lin Q, Niu Y, Luo X, Li N (2022). Determination and characterization of a novel birnavirus associated with massive mortality in Largemouth bass. Microbiology Spectrum.

[ref-66] Furuya Y, Regner M, Lobigs M, Koskinen A, Müllbacher A, Alsharifi M (2010). Effect of inactivation method on the cross-protective immunity induced by whole “killed” influenza A viruses and commercial vaccine preparations. Journal of General Virology.

[ref-67] Galanis KA, Nastou KC, Papandreou NC, Petichakis GN, Pigis DG, Iconomidou VA (2021). Linear B-cell epitope prediction for in silico vaccine design: a performance review of methods available via command-line interface. International Journal of Molecular Sciences.

[ref-68] Gao F, Liu J, Lu M, Liu Z, Wang M, Ke X, Yi M, Cao J (2021a). Nile tilapia Toll-like receptor 7 subfamily: intracellular TLRs that recruit MyD88 as an adaptor and activate the NF-kB pathway in the immune response. Developmental and Comparative Imunology.

[ref-69] Gao S, Xu T, Qiao R, Lu J, Xu Y, Hu S, Wei Y, Qi Z (2021b). Two non-mammalian toll-like receptors (TLR21 and TLR22) from golden pompano (Trachinotus ovatus): molecular cloning, gene characterizarion and expression analysis. Aquaculture Reports.

[ref-70] Geng M, Hua Y, Liu Y, Quan J, Hu X, Su P, Li Y, Liu X, Li Q, Zhu T (2021). Evolutionary history and functional characterization of Lj-TICAM-a and Lj-TICAM-b formed via lineage-specific tandem duplication in lamprey (Lampetra japonica). Genomics.

[ref-71] Geng Y, Wang KY, Huang XL, Chen DF, Li CW, Ren SY, Liao YT, Zhou ZY, Liu QF, Du ZJ, Lai WM (2012). Streptococcus agalactiae, an emerging pathogen for cultured Ya-Fish, Schizothorax prenanti, in china. Transboundary and Emerging Diseases.

[ref-72] Ghosh P, Patra P, Mondal N, Chini DS, Patra BC (2023). Multi epitopic peptide based vaccine development targeting immobilization antigen of ichthyophthirius multifiliis: a computational approach. International Journal of Peptide Research and Therapeutics.

[ref-73] Gräwe A, Ranglack J, Weyrich A, Stein V (2020). IFLinkC: an iterative functional linker cloning strategy for the combinatorial assembly and recombination of linker peptides with functional domains. Nucleic Acids Research.

[ref-74] Gries CM, Mohan RR, Morikis D, Lo DD (2019). Crosslinked flagella as a stabilized vaccine adjuvant scaffold. BMC Biotechnology.

[ref-75] Guan P, Hattotuwagama CK, Doytchinova IA, Flower DR (2006). MHCPred 2.0: an updated quantitative T-cell epitope prediction server. Applied Bioinformatics.

[ref-76] Gudding R, Van Muiswinkel WB (2013). A history of fish vaccination: science-based disease prevention in aquaculture. Fish and Shellfish Immunology.

[ref-77] Guo M, Li C (2021). An overview of cytokine used as adjuvants in fish: current state and future trends. Reviews in Aquaculture.

[ref-78] Gupta N, Regar H, Verma VK, Prusty D, Mishra A, Prajapati VK (2020). Receptor-ligand based molecular interaction to discover adjuvant for immune cell TLRs to develop next-generation vaccine. International Journal of Biological Macromolecules.

[ref-79] Gupta S, Kapoor P, Chaudhary K, Gautam A, Kumar R, Raghava GPS (2013). In silico approach for predicting toxicity of peptides and proteins. PLOS ONE.

[ref-80] Haase K, Raffegerst S, Schendel DJ, Frishman D (2015). Expitope: a web server for epitope expression. Bioinformatics.

[ref-81] Harikrishnan R, Balasundaram C, Heo M-S (2011). Fish health aspects in grouper aquaculture. Aquaculture.

[ref-82] Hazreen-Nita M, Azila A, Mukai Y, Firdaus-Nawi M, Nur-Nazifah M (2019). A review of betanodavirus vaccination as preventive strategy to viral nervous necrosis (VNN) disease in grouper. Aquaculture International.

[ref-83] He Y, Pan H, Zhang G, He S (2019a). Comparative study on pattern recognition receptors in non-teleost ray-finned fishes and their evolutionary significance in primitive vertebrates. Science China Life Sciences.

[ref-84] He L, Yu X, He J, Qiao X, Zhang Y, Lin H, Lu D (2019b). TLR5M and TLR5S play opposite roles in NF-KB pathway in Vibrio parahaemolyticus flagellin stimulation in orange-spotted grouper, Epinephelus coioides. Fish and Shellfish Immunology.

[ref-85] Heckman TI, Shahin K, Henderson EE, Griffin MJ, Soto E (2022). Development and efficacy of Streptococcus iniae live-attenuated vaccines in Nile tilapia, Oreochromis niloticus. Fish and Shellfish Immunology.

[ref-86] Hirai Y, Asahata-Tago S, Ainoda Y, Fujita T, Kikuchi K (2015). Edwardsiella tarda bacteremia, a rare but fatal water- and foodborne infection: review of the literature and clinical cases from a single centre. Canadian Journal of Infectious Diseases and Medical Microbiology.

[ref-87] Hølvold LB, Myhr AI, Dalmo RA (2014). Strategies and hurdles using DNA vaccines to fish. Veterinary Research.

[ref-88] Hobernik D, Bros M (2018). DNA vaccines—how far from clinical use?. International Journal of Molecular Sciences.

[ref-89] Hoelzer K, Bielke L, Blake DP, Cox E, Cutting SM, Devriendt B, Erlacher-Vindel E, Goossens E, Karaca K, Lemiere S, Metzner M, Raicek M, Collell Suriñach M, Wong NM, Gay C, Van Immerseel F (2018). Vaccines as alternatives to antibiotics for food producing animals, part 1: challenges and needs. Veterinary Research.

[ref-90] Honda-Okubo Y, Baldwin J, Petrovsky N (2021). Advax-CpG adjuvant provides antigen dose-sparing and enhanced immunogenicity for inactivated poliomyelitis virus vaccines. Pathogens.

[ref-91] Hu W, Jain A, Gao Y, Dozmorov IM, Mandraju R, Wakel EK, Pasare C (2015). Differential outcome of TRIF-mediated signaling in TLR4 and TLR3 induced DC maturation. Proceedings of the National Academy of Sciences of the United States of America.

[ref-92] Huang HY, Chen YC, Wang PC, Tsai MA, Yeh SC, Liang HJ, Chen SC (2014). Efficacy of a formalin-inactivated vaccine against Streptococcus iniae infection in the farmed grouper Epinephelus coioides by intraperitoneal immunization. Vaccine.

[ref-93] Huang X, Ma Y, Wang Y, Niu C, Liu Z, Yao X, Jiang X, Pan R, Jia S, Li D, Guan X, Wang L, Xu Y (2021). Oral probiotic vaccine expressing Koi herpesvirus (KHV) ORF81 protein delivered by chitosan-alginate capsules is a promising strategy for mass oral vaccination of Carps against KHV infection. Journal of Virology.

[ref-94] Irshath AA, Rajan AP, Vimal S, Prabhakaran VS, Ganesan R (2023). Bacterial pathogenesis in various fish diseases: recent advances and specific challenges in vaccine development. Vaccines 2023.

[ref-95] Islam SI, Mahfuj S, Alam MDA, Ara Y, Sanjida S, Mou MJ (2022a). Immunoinformatic approaches to identify immune epitopes and design an epitope-based subunit vaccine against emerging Tilapia lake virus (TiLV). Aquaculture Journal.

[ref-96] Islam SI, Mou MJ, Sanjida S (2022). Application of reverse vaccinology for designing of an mRNA vaccine against re-emerging marine birnavirus affecting fish species. Informatics in Medicine Unlocked.

[ref-97] Islam SI, Mou MJ, Sanjida S, Tariq M, Nasir S, Mahfuj S (2022b). Designing a novel mRNA vaccine against Vibrio harveyi infection in fish: an immunoinformatics approach. Genomics & Informatics.

[ref-98] Jensen KK, Andreatta M, Marcatili P, Buus S, Greenbaum JA, Yan Z, Sette A, Peters B, Nielsen M (2018). Improved methods for predicting peptide binding affinity to MHC class II molecules. Immunology.

[ref-99] Jespersen MC, Mahajan S, Peters B, Nielsen M, Marcatili P (2019). Antibody specific B-cell epitope predictions: leveraging information from antibody-antigen protein complexes. Frontiers in Immunology.

[ref-100] Jespersen MC, Peters B, Nielsen M, Marcatili P (2017). BepiPred-2.0: improving sequence-based B-cell epitope prediction using conformational epitopes. Nucleic Acids Research.

[ref-101] Ji J, Liao Z, Rao Y, Li W, Yang C, Yuan G, Feng H, Xu Z, Shao J, Su J (2020). Thoroughly remold the localization and signaling pathway of TLR22. Frontiers in Immunology.

[ref-102] Jiang J, Zhao W, Xiong Q, Wang K, He Y, Wang J, Chen D, Geng Y, Huang X, Ouyang P, Lai W (2017). Immune responses of channel catfish following the stimulation of three recombinant flagellins of Yersinia ruckeri in vitro and in vivo. Developmental and Comparative Immunology.

[ref-103] Jiang L, Pei L, Wang P, Liu L, Li G, Liu B, Lü Z, Hiromasa T, Pan H, Ogura A (2020). Molecular characterization and evolution analysis of two forms of TLR5 and TLR13 genes base on Larimichthys crocea genome data. International Journal of Genomics.

[ref-104] Jin S, Zhao X, Wang H, Su J, Wang J, Ding C, Li Y, Xiao T (2018). Molecular characterization and expression of TLR7 and TLR8 in barbel chub (Squaliobarbus curriculus): responses to stimulation of grass carp reovirus and lipopolysaccharide. Fish and Shellfish Immunology.

[ref-105] Joshi A, Pathak DC, Mannan ul MA, Kaushik V (2021). In-silico designing of epitope-based vaccine against the seven banded grouper nervous necrosis virus affecting fish species. Network Modeling Analysis in Health Informatics and Bioinformatics.

[ref-106] Kanwal Z, Wiegertjes GF, Veneman WJ, Meijer AH, Spaink HP (2014). Comparative studies of Toll-like receptor signalling using zebrafish. Developmental and Comparative Immunology.

[ref-107] Kar T, Narsaria U, Basak S, Deb D, Castiglione F, Mueller DM, Srivastava AP (2020). A candidate multi—epitope vaccine against SARS—CoV—2. Scientific Reports.

[ref-108] Kar P, Ruiz-Perez L, Arooj M, Mancera RL (2018). Current methods for the prediction of T-cell epitopes. Peptide Science.

[ref-109] Kaushik V (2020). In silico identification of epitope-based peptide vaccine for Nipah virus. International Journal of Peptide Research and Therapeutics.

[ref-110] Kawasaki T, Kawai T (2014). Toll-like receptor signaling pathways. Frontiers in Immunology.

[ref-111] Kayansamruaj P, Areechon N, Unajak S (2020). Development of fish vaccine in southeast asia: a challenge for the sustainability of SE asia aquaculture. Fish and Shellfish Immunology.

[ref-112] Kelley LA, Mezulis S, Yates CM, Wass MN, Sternberg MJE (2015). The Phyre2 web portal for protein modeling, prediction and analysis. Nature Protocols.

[ref-113] Kenny EF, O’Neill LAJ (2008). Signalling adaptors used by toll-like receptors: an update. Cytokine.

[ref-114] Khan S, Ullah MW, Siddique R, Nabi G, Manan S, Yousaf M, Hou H (2016). Role of recombinant DNA technology to improve life. International Journal of Genomics.

[ref-115] Kim S, Oshima H, Zhang H, Kern NR, Re S, Lee J, Roux B, Sugita Y, Jiang W, Im W (2020). CHARMM-GUI free energy calculator for absolute and relative ligand solvation and binding free energy simulations. Journal of Chemical Theory and Computation.

[ref-116] Kohnke B, Kutzner C, Grubmüller H (2020). A GPU-accelerated fast multipole method for GROMACS: performance and accuracy. Journal of Chemical Theory and Computation.

[ref-117] Krishnaswamy GT, Gururaj P, Gupta R, Gopal DR, Rajesh P, Chidambaram B, Kalyanasundaram A, Angamuthu R (2014). Transcriptome profiling reveals higher vertebrate orthologous of intra-cytoplasmic pattern recognition receptors in grey bamboo shark. PLOS ONE.

[ref-118] Kumar D, Prasad Y, Singh AK, Ansari A (2012). Columnaris disease and its drug resistance in cultured exotic african catfish Clarias gariepinus in India. Biochemical and Cellular Archives.

[ref-119] Kumru OS, Joshi SB, Smith DE, Middaugh CR, Prusik T (2014). Vaccine instability in the cold chain: mechanisms, analysis and formulation strategies. Biologicals.

[ref-120] Kuntal BK, Aparoy P, Reddanna P (2010). EasyModeller: a graphical interface to MODELLER. BMC Research Notes.

[ref-121] Larsen MV, Lundegaard C, Lamberth K, Buus S, Lund O, Nielsen M (2007). Large-scale validation of methods for cytotoxic T-lymphocyte epitope prediction. BMC Bioinformatics.

[ref-122] Lee N-H, Lee J-A, Park S-Y, Song C-S, Choi I-S, Lee J-B (2012). A review of vaccine development and research for industry animals in Korea. Clinical and Experimental Vaccine Research.

[ref-123] Leman JK, Weitzner BD, Lewis SM, Adolf-Bryfogle J, Alam N, Alford RF, Aprahamian M, Baker D, Barlow KA, Barth P, Basanta B, Bender BJ, Blacklock K, Bonet J, Boyken SE, Bradley P, Bystroff C, Conway P, Cooper S, Correia BE, Coventry B, Das R, de Jong RM, DiMaio F, Dsilva L, Dunbrack R, Ford AS, Frenz B, Fu DY, Geniesse C, Goldschmidt L, Gowthaman R, Gray JJ, Gront D, Guffy S, Horowitz S, Huang PS, Huber T, Jacobs TM, Jeliazkov JR, Johnson DK, Kappel K, Karanicolas J, Khakzad H, Khar KR, Khare SD, Khatib F, Khramushin A, King IC, Kleffner R, Koepnick B, Kortemme T, Kuenze G, Kuhlman B, Kuroda D, Labonte JW, Lai JK, Lapidoth G, Leaver-Fay A, Lindert S, Linsky T, London N, Lubin JH, Lyskov S, Maguire J, Malmström L, Marcos E, Marcu O, Marze NA, Meiler J, Moretti R, Mulligan VK, Nerli S, Norn C, Ó’Conchúir S, Ollikainen N, Ovchinnikov S, Pacella MS, Pan X, Park H, Pavlovicz RE, Pethe M, Pierce BG, Pilla KB, Raveh B, Renfrew PD, Burman SSR, Rubenstein A, Sauer MF, Scheck A, Schief W, Schueler-Furman O, Sedan Y, Sevy AM, Sgourakis NG, Shi L, Siegel JB, Silva DA, Smith S, Song Y, Stein A, Szegedy M, Teets FD, Thyme SB, Wang RYR, Watkins A, Zimmerman L, Bonneau R (2020). Macromolecular modeling and design in Rosetta: recent methods and frameworks. Nature Methods.

[ref-124] Lemoine CH, Nidom RV, Ventura R, Indrasari S, Normalina I, Santoso KP, Derouet F, Barnier-Quer C, Borchard G, Collin N, Nidom CA (2021). Better pandemic influenza preparedness through adjuvant technology transfer: challenges and lessons learned. Vaccine.

[ref-125] Leung D, Wurst J, Liu T, Martinez R, Datta-Mannan A, Feng Y (2020). Antibody conjugates-recent advances and future innovations. Antibodies.

[ref-126] Li Y, Han R, Wang J, Yang M, Dan X, Li A (2018). Molecular identification and functional characterization of IRAK-3 from a teleost fish, the orange-spotted grouper (Epinephelus coioides). Fish and Shellfish Immunology.

[ref-127] Li Y, Li Y, Cao X, Jin X, Jin T (2017). Pattern recognition receptors in zebrafish provide functional and evolutionary insight into innate immune signaling pathways. Cellular and Molecular Immunology.

[ref-128] Liang S, Zheng D, Standley DM, Yao B, Zacharias M, Zhang C (2010). EPSVR and EPMeta: prediction of antigenic epitopes using support vector regression and multiple server results. BMC Bioinformatics.

[ref-129] Liang S, Zheng D, Zhang C, Zacharias M (2009). Prediction of antigenic epitopes on protein surfaces by consensus scoring. BMC Bioinformatics.

[ref-130] Liang Z, Zhu H, Wang X, Jing B, Li Z, Xia X, Sun H, Yang Y, Zhang W, Shi L, Zeng H, Sun B (2020). Adjuvants for coronavirus vaccines. Frontiers in Immunology.

[ref-131] Liao Z, Wan Q, Su H, Wu C, Su J (2017). Pattern recognition receptors in grass carp Ctenopharyngodon idella: I. organization and expression analysis of TLRs and RLRs. Developmental and Comparative Immunology.

[ref-132] Liu KH, Ascenzi MA, Bellezza CA, Bezuidenhout AJ, Cote PJ, Gonzalez-Aseguinolaza G, Hannaman D, Luxembourg A, Evans CF, Tennant BC, Menne S (2011). Electroporation enhances immunogenicity of a DNA vaccine expressing woodchuck hepatitis virus surface antigen in woodchucks. Journal of Virology.

[ref-133] Liu R, Liu X, Song M, Qi Y, Li H, Yang G, Shan S (2021). Cyprinus carpio TRIF participates in the innate immune response by inducing NF-*κ*B and IFN activation and promoting apoptosis. Frontiers in Immunology.

[ref-134] Liu R, Qi Y, Feng H, Niu Y, Zhang F, Yang G, Shan S (2022). Fish-specific Toll-like receptor 14 (TLR14) from Asian swamp eel (Monopterus albus) is involved in immune response to bacterial infection. Fish and Shellfish Immunology.

[ref-135] Loes AN, Hinman MN, Farnsworth DR, Miller AC, Guillemin K, Harms MJ (2021). Identification and characterization of zebrafish Tlr4 coreceptor Md-2. The Journal of Immunology.

[ref-136] Lorenzen N, LaPatra SE (2005). DNA vaccines for aquacultured fish. Revue Scientifique Et Technique.

[ref-137] Loring HS, Thompson PR (2020). Emergence of SARM1 as a potential therapeutic target for Wallerian-type diseases. Cell Chemical Biology.

[ref-138] Lundegaard C, Lund O, Nielsen M (2012). Predictions versus high-throughput experiments in T-cell epitope discovery: competition or synergy?. Expert Review of Vaccines.

[ref-139] Luo L, Lucas RM, Liu L, Stow JL (2020). Signalling, sorting and scaffolding adaptors for Toll-like receptors. Journal of Cell Science.

[ref-140] Ma J, Bruce TJ, Jones EM, Cain KD (2019). A review of fish vaccine development strategies: conventional methods and modern biotechnological approaches. Microorganisms.

[ref-141] MacCarthy EA, Zhang C, Zhang Y, DB KC (2022). GPU-I-TASSER: a GPU accelerated I-TASSER protein structure prediction tool. Bioinformatics.

[ref-142] Machimbirike VI, Pornputtapong N, Senapin S, Wangkahart E, Srisapoome P, Khunrae P, Rattanarojpong T (2022a). A multi-epitope chimeric protein elicited a strong antibody response and partial protection against Edwardsiella ictaluri in Nile tilapia. Journal of Fish Diseases.

[ref-143] Machimbirike VI, Pornputtapong N, Senapin S, Wangkahart E, Srisapoome P, Khunrae P, Rattanarojpong T (2022b). A multi-epitope chimeric protein elicited a strong antibody response and partial protection against Edwardsiella ictaluri in Nile tilapia. Journal of Fish Diseases.

[ref-144] Maeda DLNF, Tian D, Yu H, Dar N, Rajasekaran V, Meng S, Mahsoub HM, Sooryanarain H, Wang B, Heffron CL, Hassebroek A, LeRoith T, Meng X-J, Zeichner SL (2021). Killed whole-genome reduced-bacteria surface-expressed coronavirus fusion peptide vaccines protect against disease in a porcine model. Proceedings of the National Academy of Sciences of the United States of America.

[ref-145] Magadan S, Sunyer JO, Boudinot P (2015). Unique features of fish immune repertoires: particularities of adaptive immunity within the largest group of vertebrates. Results and Problems in Cell Differentiation.

[ref-146] Magnan CN, Zeller M, Kayala MA, Vigil A, Randall A, Felgner PL, Baldi P (2010). High-throughput prediction of protein antigenicity using protein microarray data. Bioinformatics.

[ref-147] Mahardika K, Mastuti I, Muzaki A, Sudewi (2016). Addition of adjuvants in recombinant subunit vaccines for the prevention of grouper sleepy disease iridovirus (GSDIV) infection in Humpback grouper, Cromileptes altivelis. Indonesian Aquaculture Journal.

[ref-148] Mahendran R, Jeyabaskar S, Sitharaman G, Michael RD, Paul AV (2016a). Computer-aided vaccine designing approach against fish pathogens Edwardsiella tarda and Flavobacterium columnare using bioinformatics softwares. Drug Design, Development and Therapy.

[ref-149] Mahendran R, Jeyabaskar S, Sitharaman G, Michael RD, Paul AV (2016b). Computer-aided vaccine designing approach against fish pathogens Edwardsiella tarda and Flavobacterium columnare using bioinformatics softwares. Drug Design, Development and Therapy.

[ref-150] Mair GC, Halwart M, Derun Y, Costa-Pierce BA (2023). A decadal outlook for global aquaculture. Journal of the World Aquaculture Society.

[ref-151] Malik AA, Ojha SC, Schaduangrat N, Nantasenamat C (2022). ABCpred: a webserver for the discovery of acetyl- and butyryl-cholinesterase inhibitors. Molecular Diversity.

[ref-152] Mancheva K, Danova S, Vilhelmova-Ilieva N, Dobreva L, Kostova K, Simeonova L, Atanasov G (2021). Viral pathogens with economic impact in aquaculture. Acta Microbiologica Bulgarica.

[ref-153] Marana MH, Von Gersdorff Jø rgensen L, Skov J, Chettri JK, Mattsson AH, Dalsgaard I, Kania PW, Buchmann K (2017). Subunit vaccine candidates against Aeromonas salmonicida in rainbow trout Oncorhynchus mykiss. PLOS ONE.

[ref-154] Marshall JS, Warrington R, Watson W, Kim HL (2018). An introduction to immunology and immunopathology. Allergy, Asthma & Clinical Immunology.

[ref-155] Martin SJ, Chen IJ, Chan AWE, Foloppe N (2020). Modelling the binding mode of macrocycles: docking and conformational sampling. Bioorganic and Medicinal Chemistry.

[ref-156] Martínez-Flores D, Zepeda-Cervantes J, Cruz-Reséndiz A, Aguirre-Sampieri S, Sampieri A, Vaca L (2021). SARS-CoV-2 vaccines based on the spike glycoprotein and implications of new viral variants. Frontiers in Immunology.

[ref-157] Marze NA, Roy Burman SS, Sheffler W, Gray JJ (2018). Efficient flexible backbone protein-protein docking for challenging targets. Bioinformatics.

[ref-158] Maurer-Stroh S, Krutz NL, Kern PS, Gunalan V, Nguyen MN, Limviphuvadh V, Eisenhaber F, Gerberick GF (2019). AllerCatPro—prediction of protein allergenicity potential from the protein sequence. Bioinformatics.

[ref-159] Miccoli A, Manni M, Picchietti S, Scapigliati G (2021). State-of-the-art vaccine research for aquaculture use: the case of three economically relevant fish species. Vaccine.

[ref-160] Michel-Todó L, Bigey P, Reche PA, Pinazo MJ, Gascón J, Alonso-Padilla J (2020). Design of an epitope-based vaccine ensemble for animal trypanosomiasis by computational methods. Vaccine.

[ref-161] Miniero Davies Y, Xavier de Oliveira MG, Paulo Vieira Cunha M, Soares Franco L, Pulecio Santos SL, Zanolli Moreno L, Túlio de Moura Gomes V, Zanolli Sato MI, Schiavo Nardi M, Micke Moreno A, Becker Saidenberg A, Rose Marques de Sá L, Knöbl T (2018). Edwardsiella tarda outbreak affecting fishes and aquatic birds in Brazil. Veterinary Quarterly.

[ref-162] Mishra A, Nam GH, Gim JA, Lee H-E, Jo A, Kim H-S (2018). Current challenges of Streptococcus infection and effective molecular, cellular, and environmental control methods in aquaculture. Molecules and Cells.

[ref-163] Mitchell CD, Criscitiello MF (2020). Comparative study of cartilaginous fish divulges insights into the early evolution of primary, secondary and mucosal lymphoid tissue architecture. Fish and Shellfish Immunology.

[ref-164] Mitiku MA (2018). A review on columnaris disease in freshwater farmed fish. International Journal of Advances Research in Biological Sciences.

[ref-165] Mohd-Aris A, Muhamad-Sofie MHN, Zamri-Saad M, Daud HM, Yasin Ina-Salwany M (2019). Live vaccines against bacterial fish diseases: a review. Veterinary World.

[ref-166] Morales-Hernández S, Ugidos-Damboriena N, López-Sagaseta J (2022). Self-assembling protein nanoparticles in the design of vaccines: 2022 update. Vaccines 2022.

[ref-167] Muduli C, Paria A, Srivastava R, Rathore G, Lal KK (2021). Aeromonas hydrophila infection induces Toll-like receptor 2 (tlr2) and associated downstream signaling in Indian catfish, Clarias magur (Hamilton, 1822). PeerJ.

[ref-168] Mugunthan SP, Harish MC (2021). Multi-epitope-based vaccine designed by targeting cytoadherence proteins of Mycoplasma gallisepticum. ACS Omega.

[ref-169] Mugwanya M, Dawood MAO, Kimera F, Sewilam H (2022). Anthropogenic temperature fluctuations and their effect on aquaculture: a comprehensive review. Agriculture and Fisheries.

[ref-170] Muktar Y, Tesfaye S (2016). Present status and future prospects of fish vaccination: a review. Journal of Veterinary Science & Technology.

[ref-171] Munangándu HM, Salinas I, Tafalla C, Dalmo RA (2020). Editorial: vaccines and immunostimulants for finfish. Frontiers in Immunology.

[ref-172] Muñoz Atienza E, Díaz-Rosales P, Tafalla C (2021). Systemic and mucosal B and T Cell responses upon mucosal vaccination of teleost fish. Frontiers in Immunology.

[ref-173] Mzula A, Wambura PN, Mdegela RH, Shirima GM (2019). Current state of modern biotechnological-based Aeromonas hydrophila vaccines for aquaculture: a systematic review. BioMed Research International.

[ref-174] Nagpal G, Chaudhary K, Agrawal P, Raghava GPS (2018). Computer-aided prediction of antigen presenting cell modulators for designing peptide-based vaccine adjuvants. Journal of Translational Medicine.

[ref-175] Nagpal G, Gupta S, Chaudhary K, Kumar Dhanda S, Prakash S, Raghava GPS (2015). VaccineDA: prediction, design and genome-wide screening of oligodeoxynucleotide-based vaccine adjuvants. Scientific Reports.

[ref-176] Nakahira Y, Mizuno K, Yamashita H, Tsuchikura M, Takeuchi K, Shiina T, Kawakami H (2021). Mass production of virus-like particles using chloroplast genetic engineering for highly immunogenic oral vaccine against fish disease. Frontiers in Plant Science.

[ref-177] Nascimento IP, Leite LCC (2012). Recombinant vaccines and the development of new vaccine strategies. Brazilian Journal of Medical and Biological Research.

[ref-178] Naz A, Shahid F, Butt TT, Awan FM, Ali A, Malik A (2020). Designing multi-epitope vaccines to combat emerging coronavirus disease 2019 (COVID-19) by employing immuno-informatics approach. Frontiers in Immunology.

[ref-179] Nie L, Cai SY, Shao JZ, Chen J (2018). Toll-Like receptors, associated biological roles, and signaling networks in non-mammals. Frontiers in Immunology.

[ref-180] Ogishi M, Yotsuyanagi H (2019). Quantitative prediction of the landscape of T cell epitope immunogenicity in sequence space. Frontiers in Immunology.

[ref-181] Olshefsky A, Richardson C, Pun SH, King NP (2022). Engineering self-assembling protein nanoparticles for therapeutic delivery. Bioconjugate Chemistry.

[ref-182] Palgen JL, Feraoun Y, Dzangué-Tchoupou G, Joly C, Martinon F, Le Grand R, Beignon AS (2021). Optimize prime/boost vaccine strategies: trained immunity as a new player in the game. Frontiers in Immunology.

[ref-183] Palti Y (2011). Toll-like receptors in bony fish: from genomics to function. Developmental and Comparative Immunology.

[ref-184] Pambudi NA, Sarifudin A, Gandidi IM, Romadhon R (2022). Vaccine cold chain management and cold storage technology to address the challenges of vaccination programs. Energy Reports.

[ref-185] Pang Y (2016). FF12MC: a revised AMBER forcefield and new protein simulation protocol. Proteins: Structure, Function, and Bioinformatics.

[ref-186] Paria A, Makesh M, Chaudhari A, Purushothaman CS, Rajendran KV (2018). Toll-like receptor (TLR) 22, a non-mammalian TLR in Asian seabass, Lates calcarifer: characterisation, ontogeny and inductive expression upon exposure with bacteria and ligands. Developmental and Comparative Immunology.

[ref-187] Park SB, Aoki T, Jung TS (2012). Pathogenesis of and strategies for preventing Edwardsiella tarda infection in fish. Veterinary Research.

[ref-188] Park H, Kim DE, Ovchinnikov S, Baker D, DiMaio F (2018). Automatic structure prediction of oligomeric assemblies using Robetta in CASP12. Proteins: Structure, Function, and Bioinformatics.

[ref-189] Parra D, Reyes-Lopez FE, Tort L (2015). Mucosal immunity and B cells in teleosts: effect of vaccination and stress. Frontiers in Immunology.

[ref-190] Patronov A, Doytchinova I (2013). T-cell epitope vaccine design by immunoinformatics. Open Biology.

[ref-191] Peignier A, Parker D (2020). Trained immunity and host-pathogen interactions. Cellular Microbiology.

[ref-192] Peng J, Yuan Q, Lin B, Panneerselvam P, Wang X, Luan XL, Lim SK, Leung BP, Ho B, Ding JL (2010). SARM inhibits both TRIF- and MyD88-mediated AP-1 activation. European Journal of Immunology.

[ref-193] Pereira UP, Soares SC, Blom J, Leal CAG, Ramos RTJ, Guimarães LC, Oliveira LC, Almeida SS, Hassan SS, Santos AR, Miyoshi A, Silva A, Tauch A, Barh D, Azevedo V, Figueiredo HCP (2013). In silico prediction of conserved vaccine targets in Streptococcus agalactiae strains isolated from fish, cattle, and human samples. Genetics and Molecular Research.

[ref-194] Peterman MA, Posadas BC (2019). Direct economic impact of fish diseases on the East Mississippi catfish industry. North American Journal of Aquaculture.

[ref-195] Phillips JC, Hardy DJ, Maia JDC, Stone JE, Ribeiro JV, Bernardi RC, Buch R, Fiorin G, Hénin J, Jiang W, McGreevy R, Melo MCR, Radak BK, Skeel RD, Singharoy A, Wang Y, Roux B, Aksimentiev A, Luthey-Schulten Z, Kalé LV, Schulten K, Chipot C, Tajkhorshid E (2020). Scalable molecular dynamics on CPU and GPU architectures with NAMD. Journal of Chemical Physics.

[ref-196] Ponomarenko J, Bui HH, Li W, Fusseder N, Bourne PE, Sette A, Peters B (2008). ElliPro: a new structure-based tool for the prediction of antibody epitopes. BMC Bioinformatics.

[ref-197] Poolman J, Borrow R (2011). Hyporesponsiveness and its clinical implications after vaccination with polysaccharide or glycoconjugate vaccines. Expert Review of Vaccines.

[ref-198] Poreba M (2020). Protease-activated prodrugs: strategies, challenges, and future directions. FEBS Journal.

[ref-199] Preena PG, Dharmaratnam A, Swaminathan TR (2022). A peek into mass mortality caused by antimicrobial resistant Edwardsiella tarda in goldfish, Carassius auratus in Kerala. Biologia.

[ref-200] Priyam M, Gupta SK, Sarkar B, Sharma TR, Pattanayak A (2020). Variation in selection constraints on teleost TLRs with emphasis on their repertoire in the Walking catfish, Clarias batrachus. Scientific Reports.

[ref-201] Prosser W, Spisak C, Hatch B, McCord J, Tien M, Roche G (2021). Designing supply chains to meet the growing need of vaccines: evidence from four countries. Journal of Pharmaceutical Policy and Practice.

[ref-202] Pumchan A, Krobthong S, Roytrakul S, Sawatdichaikul O, Kondo H, Hirono I, Areechon N, Unajak S (2020). Novel chimeric multiepitope vaccine for streptococcosis disease in Nile tilapia (Oreochromis niloticus Linn.). Scientific Reports.

[ref-203] Qi Z, Wang S, Zhu X, Yang Y, Han P, Zhang Q, Zhang S, Shao R, Xu Q, Wei Q (2018). Molecular characterization of three toll-like receptors (TLR21, TLR22, and TLR25) from a primitive ray-finned fish Dabry’s sturgeon (Acipenser dabryanus). Fish and Shellfish Immunology.

[ref-204] Radwan J, Babik W, Kaufman J, Lenz TL, Winternitz J (2020). Advances in the evolutionary understanding of MHC polymorphism. Trends in Genetics.

[ref-205] Rauta PR, Samanta M, Dash HR, Nayak B, Das S (2014). Toll-like receptors (TLRs) in aquatic animals: signaling pathways, expressions and immune responses. Immunology Letters.

[ref-206] Razali SA, Sarah Diana P, Shamsir MS, Mahadi NM, Mohd Illias R (2016). Substrate and cofactor binding interaction studies of galactitol -1- Phosphate 5- Dehydrogenase from Peptoclostridium difficile. Jurnal Teknologi.

[ref-207] Razali SA, Shamsir MS (2020). Characterisation of a catalytic triad and reaction selectivity in the dual mechanism of the catalyse hydride transfer in xylitol phosphate dehydrogenase. Journal of Molecular Graphics and Modelling.

[ref-208] Rebl A, Goldammer T, Seyfert HM (2010). Toll-like receptor signaling in bony fish. Veterinary Immunology and Immunopathology.

[ref-209] Rebl A, Rebl H, Verleih M, Haupt S, Köbis JM, Goldammer T, Seyfert HM (2019). At Least two genes encode many variants of Irak3 in rainbow trout, but neither the full-length factor nor its variants. Frontiers in Immunology.

[ref-210] Reche PA, Glutting JP, Zhang H, Reinherz EL (2004). Enhancement to the RANKPEP resource for the prediction of peptide binding to MHC molecules using profiles. Immunogenetics.

[ref-211] Reynisson B, Alvarez B, Paul S, Peters B, Nielsen M (2020). NetMHCpan-4.1 and NetMHCIIpan-4.0: improved predictions of MHC antigen presentation by concurrent motif deconvolution and integration of MS MHC eluted ligand data. Nucleic Acids Research.

[ref-212] Ringø E, Olsen RE, Jensen I, Romero J, Lauzon HL (2014). Application of vaccines and dietary supplements in aquaculture: possibilities and challenges. Reviews in Fish Biology and Fisheries.

[ref-213] Rodrigues CMC, Plotkin SA (2020). Impact of vaccines; health, economic and social perspectives. Frontiers in Microbiology.

[ref-214] Roel-Touris J, Don CG, Honorato RR, Rodrigues JPGLM, Bonvin AMJJ (2019). Less is more: coarse-grained integrative modeling of large biomolecular assemblies with HADDOCK. Journal of Chemical Theory and Computation.

[ref-215] Rubinstein ND, Mayrose I, Martz E, Pupko T (2009). Epitopia: a web-server for predicting B-cell epitopes. BMC Bioinformatics.

[ref-216] Rudra JS, Tian YF, Jung JP, Collier JH (2010). A self-assembling peptide acting as an immune adjuvant. Proceedings of the National Academy of Sciences of the United States of America.

[ref-217] Saadi M, Karkhah A, Nouri HR (2017). Development of a multi-epitope peptide vaccine inducing robust T cell responses against brucellosis using immunoinformatics based approaches. Infection, Genetics and Evolution.

[ref-218] Sami SA, Marma KKS, Mahmud S, Khan MAN, Albogami S, El-Shehawi AM, Rakib A, Chakraborty A, Mohiuddin M, Dhama K, Uddin MMN, Hossain MK, Tallei TE, Emran T Bin (2021). Designing of a multi-epitope vaccine against the structural proteins of Marburg virus exploiting the immunoinformatics approach. ACS Omega.

[ref-219] Sanches RCO, Tiwari S, Ferreira LCG, Oliveira FM, Lopes MD, Passos MJF, Maia EHB, Taranto AG, Kato R, Azevedo VAC, Lopes DO (2021). Immunoinformatics design of multi-epitope peptide-based vaccine against Schistosoma mansoni using transmembrane proteins as a target. Frontiers in Immunology.

[ref-220] Sanchez-Trincado JL, Gomez-Perosanz M, Reche PA (2017). Fundamentals and methods for T- and B-cell epitope prediction. Journal of Immunology Research.

[ref-221] Sanders B, Koldijk M, Schuitemaker H, Nunnally B, Turula V, Sitrin R (2015). Inactivated viral vaccines. Vaccine analysis: strategies, principles, and control.

[ref-222] Santos-Martins D, Solis-Vasquez L, Tillack AF, Sanner MF, Koch A, Forli S (2021). Accelerating A uto D ock 4 with GPUs and gradient-based local search. Journal of Chemical Theory and Computation.

[ref-223] Sayers S, Ulysse G, Xiang Z, He Y (2012). Vaxjo: a web-based vaccine adjuvant database and its application for analysis of vaccine adjuvants and their uses in vaccine development. Journal of Biomedicine and Biotechnology.

[ref-224] Shanmugam A, Rajoria S, George AL, Mittelman A, Suriano R, Tiwari RK (2012). Synthetic toll like receptor-4 (TLR-4) agonist peptides as a novel class of adjuvants. PLOS ONE.

[ref-225] Sharma G, Holt RA (2014). T-cell epitope discovery technologies. Human Immunology.

[ref-226] Sharma N, Patiyal S, Dhall A, Pande A, Arora C, Raghava GPS (2021). AlgPred 2.0: an improved method for predicting allergenic proteins and mapping of IgE epitopes. Briefings in Bioinformatics.

[ref-227] Shefat SHT (2018). Vaccines for use in finfish aquaculture. Acta Scientific Pharmaceutical Sciences.

[ref-228] Shoemaker CA, Klesius PH, Drennan JD, Evans JJ (2011). Efficacy of a modified live Flavobacterium columnare vaccine in fish. Fish and Shellfish Immunology.

[ref-229] Shoemaker CA, Klesius PH, Evans JJ, Arias CR (2009). Use of modified live vaccines in aquaculture. Journal of the World Aquaculture Society.

[ref-230] Šimková A, Gettová L, Civáňová K, Seifertová M, Janáč M, Vetešník L (2021). Diversity of MHC IIB genes and parasitism in hybrids of evolutionarily divergent cyprinoid species indicate heterosis advantage. Scientific Reports.

[ref-231] Singh H, Ansari HR, Raghava GPS (2013). Improved method for linear B-cell epitope prediction using antigen’s primary sequence. PLOS ONE.

[ref-232] Singh H, Raghava GPS (2001). ProPred: prediction of HLA-DR binding sites. Bioinformatics Applications Note.

[ref-233] Singh H, Raghava GPS (2003). ProPred1: prediction of promiscuous MHC Class-I binding sites. Bioinformatics (Oxford, England).

[ref-234] Sirimanapong W, Thompson KD, Shinn AP, Adams A, Withyachumnarnkul B (2018). Streptococcus agalactiae infection kills red tilapia with chronic Francisella noatunensis infection more rapidly than the fish without the infection. Fish and Shellfish Immunology.

[ref-235] Smith NC, Rise ML, Christian SL (2019). A comparison of the innate and adaptive immune systems in cartilaginous fish, ray-finned fish, and lobe-finned fish. Frontiers in Immunology.

[ref-236] Sneeringer S, Bowman M, Clancy M (2019). The U.S. and EU animal pharmaceutical industries in the age of antibiotic resistance. https://www.ers.usda.gov/publications/pub-details/?pubid=93178.

[ref-237] Sommerset I, Krossøy B, Biering E, Frost P (2005). Vaccines for fish in aquaculture. Expert Review of Vaccines.

[ref-238] Soria-Guerra RE, Nieto-Gomez R, Govea-Alonso DO, Rosales-Mendoza S (2015). An overview of bioinformatics tools for epitope prediction: implications on vaccine development. Journal of Biomedical Informatics.

[ref-239] Sousa C, Fernandes SA, Cardoso JCR, Wang Y, Zhai W, Guerreiro PM, Chen L, Canário AVM, Power DM (2022). Toll-like receptor evolution: does temperature matter?. Frontiers in Immunology.

[ref-240] Palatnik-de Sousa CB, Soares I da S, Rosa DS (2018). Editorial: epitope discovery and synthetic vaccine design. Frontiers in Immunology.

[ref-241] Su YL, Feng J, Li YW, Bai JS, Li AX (2016). Development of a quantitative PCR assay for monitoring Streptococcus agalactiae colonization and tissue tropism in experimentally infected Tilapia. Journal of Fish Diseases.

[ref-242] Sudheesh PS, Al-Ghabshi A, Al-Mazrooei N, Al-Habsi S (2012). Comparative pathogenomics of bacteria causing infectious diseases in fish. International Journal of Evolutionary Biology.

[ref-243] Sudheesh PS, Cain KD (2017). Prospects and challenges of developing and commercializing immersion vaccines for aquaculture. International Biology Review.

[ref-244] Sun J, Wu D, Xu T, Wang X, Xu X, Tao L, Li YX, Cao ZW (2009). SEPPA: a computational server for spatial epitope prediction of protein antigens. Nucleic Acids Research.

[ref-245] Sweredoski MJ, Baldi P (2008). PEPITO: improved discontinuous B-cell epitope prediction using multiple distance thresholds and half sphere exposure. Bioinformatics.

[ref-246] Tacchi L, Misra M, Salinas I (2013). Anti-viral immune responses in a primitive lung: characterization and expression analysis of interferon-inducible immunoproteasome subunits LMP2, LMP7 and MECL-1 in a sarcopterygian fish, the Nigerian spotted lungfish (Protopterus dolloi). Developmental and Comparative IImunology.

[ref-247] Tafalla C, Bø gwald J, Dalmo RA (2013). Adjuvants and immunostimulants in fish vaccines: current knowledge and future perspectives. Fish and Shellfish Immunology.

[ref-248] Tajimi S, Kondo M, Nakanishi T, Nagasawa T, Nakao M, Somamoto T (2019). Generation of virus-specific CD8+ T cells by vaccination with inactivated virus in the intestine of ginbuna crucian carp. Developmental and Comparative Immunology.

[ref-249] Tavares-Dias M, Martins ML (2017). An overall estimation of losses caused by diseases in the Brazilian fish farms. Journal of Parasitic Diseases: Official Organ of the Indian Society for Parasitology.

[ref-250] Tomar N, De RK, De RK, Tomar N (2014). Immunoinformatics: a brief review. Immunoinformatics. Methods in molecular biology (methods and protocols).

[ref-251] Tong JC, Ren EC (2009). Immunoinformatics: current trends and future directions. Drug Discovery Today.

[ref-252] Toranzo AE, Magariños B, Romalde JL (2005). A review of the main bacterial fish diseases in mariculture systems. Aquaculture.

[ref-253] Troutman TD, Bazan JF, Pasare C (2012). Toll-like receptors, signaling adapters and regulation of the pro-inflammatory response by PI3K. Cell Cycle.

[ref-254] Vaughn DW, Whitehead SS, Durbin AP, Barrett ADT, Stanberry LR (2009). Chapter 19 - Dengue. Vaccines for biodefense and emerging and neglected diseases.

[ref-255] Vita R, Mahajan S, Overton JA, Dhanda SK, Martini S, Cantrell JR, Wheeler DK, Sette A, Peters B (2019). The immune epitope database (IEDB): 2018 update. Nucleic Acids Research.

[ref-256] Vreven T, Vangaveti S, Borrman TM, Gaines JC, Weng Z (2020). Performance of ZDOCK and IRAD in CAPRI rounds 39-45. Proteins: Structure, Function and Bioinformatics.

[ref-257] Wali A, Balkhi M (2016). Fish vaccination and therapeutics. International Journal of Multidisciplinary Research and Develoment.

[ref-258] Wang KL, Chen SN, Huo HJ, Nie P (2021). Identification and expression analysis of sixteen Toll-like receptor genes, TLR1, TLR2a, TLR2b, TLR3, TLR5M, TLR5S, TLR7-9, TLR13a-c, TLR14, TLR21-23 in mandarin fish Siniperca chuatsi. Developmental and Comparative Immunology.

[ref-259] Wang N, Chen M, Wang T (2019). Liposomes used as a vaccine adjuvant-delivery system: from basics to clinical immunization. Journal of Controlled Release.

[ref-260] Wang M, Jiang S, Wang Y (2016). Recent advances in the production of recombinant recombinant subunit vaccines in Pichia pastoris. Bioengineered.

[ref-261] Wang S, Li W, Liu S, Xu J (2016). RaptorX-Property: a web server for protein structure property prediction. Nucleic Acids Research.

[ref-262] Wang J, Zhang Z, Liu J, Li F, Chang F, Fu H, Zhao J, Yin D (2015). Structural characterization and evolutionary analysis of fish-specific TLR27. Fish and Shellfish Immunology.

[ref-263] Wangkahart E, Secombes CJ, Wang T (2019). Studies on the use of flagellin as an immunostimulant and vaccine adjuvant in fish aquaculture. Frontiers in Immunology.

[ref-264] Waterhouse A, Bertoni M, Bienert S, Studer G, Tauriello G, Gumienny R, Heer FT, de Beer TAP, Rempfer C, Bordoli L, Lepore R, Schwede T (2018). SWISS-MODEL: homology modelling of protein structures and complexes. Nucleic Acids Research.

[ref-265] Wegner KM (2008). Historical and contemporary selection of teleost MHC genes: did we leave the past behind?. Journal of Fish Biology.

[ref-266] Wieczorek M, Abualrous ET, Sticht J, Álvaro Benito M, Stolzenberg S, Noé F, Freund C (2017). Major histocompatibility complex (MHC) class I and MHC class II proteins: conformational plasticity in antigen presentation. Frontiers in Immunology.

[ref-267] Wong LL, Razali SA, Deris ZM, Danish-Daniel M, Tan MP, Nor SAM, Ma H, Min W, Yantao L, Asaduzzaman M, Sung YY, Liu Z, Sorgeloos P, Van de Peer Y, Afiqah-Aleng N (2022). Application of second-generation sequencing (SGS) and third generation sequencing (TGS) in aquaculture breeding program. Aquaculture.

[ref-268] Wu M, Guo L, Zhu K-C, Guo H-Y, Liu B-S, Zhang N, Jiang S-G, Zhang D-C (2019). Molecular characterization of Toll-like receptor 14 from golden pompano Trachinotus ovatus (Linnaeus, 1758) and its expression response to three types of pathogen-associated molecular patterns. Comparative Biochemistry and Physiology, Part B.

[ref-269] Xing J, Xu H, Tang X, Sheng X, Zhan W (2019). A DNA vaccine encoding the VAA gene of vibrio anguillarum induces a protective immune response in flounder. Frontiers in Immunology.

[ref-270] Xu Y-R, Lei C-Q (2021). TAK1-TABs complex: a central signalosome in inflammatory responses. Frontiers in Immunology.

[ref-271] Yanong RP (2017). Use of vaccines in finfish aquaculture. https://edis.ifas.ufl.edu/publication/FA156.

[ref-272] Yao B, Zhang L, Liang S, Zhang C (2012). SVMTriP: a method to predict antigenic epitopes using support vector machine to integrate Tri-peptide similarity and propensity. PLOS ONE.

[ref-273] Yi M, Wang M, Li Z, Liu Z, Song C, Zhang D, Gao F, Ke X, Cao J, Lu M (2019). An investigation into the effects of Streptococcus agalactiae on the 5-HT system and the behavior of GIFT tilapia (Oreochromis niloticus). Aquaculture Reports.

[ref-274] Yu L, Feng Z (2018). The role of Toll-like receptor signaling in the progression of heart failure. Mediators of Inflammation.

[ref-275] Zhang L (2018). Multi-epitope vaccines: a promising strategy against tumors and viral infections. Cellular and Molecular Immunology.

[ref-276] Zhang X, Chen LX, Ouyang L, Cheng Y, Liu B (2012). Plant natural compounds: targeting pathways of autophagy as anti-cancer therapeutic agents. Cell Proliferation.

[ref-277] Zhang J, Kong X, Zhou C, Li L, Nie G, Li X (2014). Toll-like receptor recognition of bacteria in fish: ligand specificity and signal pathways. Fish and Shellfish Immunology.

[ref-278] Zhang X, Liu Z, Wu S, Sun M, Wei J, Qin Q (2020). Fish RIP1 mediates innate antiviral immune responses induced by SGIV and RGNNV infection. Frontiers in Immunology.

[ref-279] Zhang Y, Liang C (2016). Innate recognition of microbial-derived signals in immunity and inflammation. Science China Life Sciences.

[ref-280] Zhou T, Yuan Z, Tan S, Jin Y, Yang Y, Shi H, Wang W, Niu D, Gao L, Jiang W, Gao D, Liu Z (2018). A review of molecular responses of catfish to bacterial diseases and abiotic stresses. Frontiers in Physiology.

[ref-281] Zhu L, Yang Q, Huang L, Wang K, Wang X, Chen D, Geng Y, Huang X, Ouyang P, Lai W (2017). Effectivity of oral recombinant DNA vaccine against Streptococcus agalactiae in Nile tilapia. Developmental and Comparative Immunology.

[ref-282] Zhu W, Yang G, Zhang Y, Yuan J, An L (2012). Generation of biotechnology-derived Flavobacterium columnare ghosts by PhiX174 gene e-mediated inactivation and the potential as vaccine candidates against infection in grass carp. Journal of Biomedicine and Biotechnology.

[ref-283] Zimmermann P, Curtis N (2019). Factors that influence the immune response to vaccination. Clinical Microbiology Reviews.

[ref-284] Zou P, Li K, Li Y, Shen Y, Zhang Z, Wang Y (2021). RIP3 associates with RIP1, TRIF, MAVS, and also IRF3/7 in host innate immune signaling in Large Yellow Croaker *Larimichthys crocea*. Antibiotics.

