# Supplementary material for: Riding the wave of innovation: immunoinformatics in fish disease control
Source: PeerJ. 2023 Dec 8;11:e16419. doi: 10.7717/peerj.16419 (PMC10712311; doi:10.7717/peerj.16419)
Supplement: Supplemental Information 1 [file peerj-11-16419-s001.docx]

**Riding the Wave of Innovation: Immunoinformatics in Fish Disease Control**

Siti Aisyah Razali^1,2*,^ Mohd Shahir Shamsir^3^, Nur Farahin Ishak^1^, Low Chen Fei^4^, Wan-Atirah Azemin^5^*

^1^Faculty of Science and Marine Environment, Universiti Malaysia Terengganu, 21030 Kuala Nerus, Terengganu, Malaysia

^2^Biological Security and Sustainability Research Interest Group (BIOSES), Universiti Malaysia Terengganu, 21030 Kuala Nerus, Terengganu, Malaysia.

^3^Department of Biosciences, Faculty of Science, Universiti Teknologi Malaysia, 81310 Skudai, Johor, Malaysia

^4^Institute of Systems Biology (INBIOSIS), Universiti Kebangsaan Malaysia, 43600 Bangi, Selangor, Malaysia

^5^School of Biological Sciences, Universiti Sains Malaysia, 11800 Minden, Pulau Pinang, Malaysia

Corresponding Author:

Siti Aisyah Razali

Faculty of Science and Marine Environment, Universiti Malaysia Terengganu, 21030 Kuala Nerus, Terengganu, Malaysia

Email address: aisyarazali@umt.edu.my

Wan-Atirah Azemin

School of Biological Sciences, Universiti Sains Malaysia, 11800 Minden, Pulau Pinang, Malaysia

Email address: wanatirah@usm.my

**Supplemental file**

**Table 1:** A summary of tools useful in immunoinformatics vaccine design for fish diseases.

| **Application** | **Method** | **Tools/ server** | **URL** |
| --- | --- | --- | --- |
| Epitope Prediction | B-cell prediction | ABCpred | https://webs.iiitd.edu.in/raghava/abcpred/ |
|  |  | IEDB B-cell epitope tools | http://tools.iedb.org/bcell/ |
|  |  | SVMTriP | http://sysbio.unl.edu/SVMTriP/index.php |
|  |  | BCPred | http://ailab-projects1.ist.psu.edu:8080/bcpred/predict.html |
|  |  | LBtope | http://crdd.osdd.net/raghava/lbtope/ |
|  |  | BepiPred 2.0 | https://services.healthtech.dtu.dk/service.php?BepiPred-2.0 |
|  |  | DiscoTope-2.0 | https://services.healthtech.dtu.dk/service.php?DiscoTope-2.0 |
|  |  | BEpro | http://pepito.proteomics.ics.uci.edu/ |
|  |  | ElliPro | http://tools.iedb.org/ellipro/ |
|  |  | EPCES | http://sysbio.unl.edu/EPCES/ |
|  |  | EPSVR | http://sysbio.unl.edu/EPSVR/ |
|  |  | EPMeta | http://sysbio.unl.edu/EPMeta/ |
|  |  | Epitopia | http://epitopia.tau.ac.il/ |
|  |  | SEPPA | http://www.badd-cao.net/seppa3/index.html |
|  | T-cell prediction | IEDB database | http://tools.immuneepitope.org/mhcii/ |
|  |  | RANKPEP server | http://imed.med.ucm.es/Tools/rankpep.HTML |
|  |  | ProPred | http://crdd.osdd.net/raghava/propred/ |
|  |  | NetMHCIIpan- 4.0 | https://services.healthtech.dtu.dk/service.php?NetMHCIIpan-4.0 |
|  |  | NetMHCIIpan- 3.2 | https://services.healthtech.dtu.dk/service.php?NetMHCIIpan-3.2 |
|  |  | NetCTL-1.2 | https://services.healthtech.dtu.dk/service.php?NetCTL-1.2 |
|  |  | ProPred1 | http://crdd.osdd.net/raghava/propred1/ |
|  |  | NetMHCpan-4.1 | https://services.healthtech.dtu.dk/service.php?NetMHCpan-4.1 |
|  |  | MHCpred 2.0 | http://www.ddg-pharmfac.net/mhcpred/MHCPred/ |
|  |  | EpiJen | http://www.ddg-pharmfac.net/epijen/EpiJen/EpiJen.htm |
|  |  | CTLPred | http://crdd.osdd.net/raghava/ctlpred/index.html |
|  |  | Expitope | http://webclu.bio.wzw.tum.de/expitope/ |
| Construction of multi-epitope vaccine | Adjuvant selection | Vaxjo | http://www.violinet.org/vaxjo/ |
|  |  | VaccineDA | https://webs.iiitd.edu.in/raghava/vaccineda/ |
|  |  | imRNA | https://webs.iiitd.edu.in/raghava/imrna/ |
|  |  | VaxinPAD | https://webs.iiitd.edu.in/raghava/vaxinpad/ |
|  | Linker selection | Linker DB | https://www.ibi.vu.nl/programs/linkerdbwww/ |
|  | Structural modeling | EasyModeller 4.0 | https://sites.google.com/site/bioinformatikz/homology-modelling/homology-modelling?authuser=0 |
|  |  | SWISS-MODEL | https://swissmodel.expasy.org/ |
|  |  | Phyre2 | http://www.sbg.bio.ic.ac.uk/~phyre2/ |
|  |  | I-TASSER | https://zhanggroup.org/I-TASSER/ |
|  |  | RaptorX | http://raptorx.uchicago.edu/ |
|  |  | ROBETTA, | http://robetta.bakerlab.org/ |
|  | Structure validation | PROCHECK | https://www.ebi.ac.uk/thornton-srv/software/PROCHECK/ |
|  |  | Verify3D | https://saves.mbi.ucla.edu |
|  |  | ERRAT | https://saves.mbi.ucla.edu |
| Prediction of vaccine antigenicity, allergenicity and toxicity | Antigenicity | VaxiJen 2.0 | http://www.ddg-pharmfac.net/vaxijen/VaxiJen/VaxiJen.html |
|  |  | ANTIGENpro | http://scratch.proteomics.ics.uci.edu/ |
|  | Allergenicity | AllerTOP v 2.0 | http://www.ddg-pharmfac.net/AllerTOP/ |
|  |  | AlgPred | https://webs.iiitd.edu.in/raghava/algpred/submission.html |
|  |  | AllerCatPro | https://allercatpro.bii.a-star.edu.sg/ |
|  | Toxicity | ToxinPred | http://crdd.osdd.net/raghava/toxinpred/ |
|  |  | T3DB | http://www.t3db.ca/biodb/search/target  _bonds/sequence |
|  | Physicochemical properties | Protparam | https://web.expasy.org/protparam/ |
| Molecular interaction of immunogenic vaccine | Molecular docking | Autodock Vina | https://vina.scripps.edu/ |
|  |  | Autodock 4 | https://autodock.scripps.edu/ |
|  |  | ZDOCK | https://zdock.umassmed.edu/ |
|  |  | Glide | https://www.schrodinger.com/products/glide |
|  |  | GOLD | https://www.ccdc.cam.ac.uk/solutions/csd-discovery/components/gold/ |
|  |  | RosettaDock | https://rosie.graylab.jhu.edu/ |
|  |  | ClusPro | https://cluspro.org/login.php |
|  |  | HADDOCK | https://wenmr.science.uu.nl/haddock2.4/ |
|  |  | EpiDOCK | http://www.ddg-pharmfac.net/epidock/EpiDockPage.html |
|  | Molecular dynamics simulation | GROMACS | https://www.gromacs.org/ |
|  |  | CHARMM | https://www.charmm.org/charmm/ |
|  |  | AMBER | http://ambermd.org/ |
|  |  | NAMD | https://www.ks.uiuc.edu/Research/namd/ |
